# Biosynthesis of Silver Nanoparticles Using Black Pepper (*Piper nigrum*) Seed Extract and Evaluation of Their Cytotoxicity

**DOI:** 10.1002/cbdv.202503359

**Published:** 2026-01-31

**Authors:** Nylton Ferreira Maciel, Gleice VasconcelosPereira do Lago, Glauce Vasconcelos da Silva Pereira, Ingryd Nayara de Farias Ramos, Aline Costa Bastos, Igor Alexandre Rocha Barreto, Nathiel Sarges Moraes, Marcos NazarenoLauné dos Santos, João Augusto Pereira da Rocha, Alencar Kolinski Machado, Waldomiro Gomes Paschoal Junior, Marcio Marcelo da Silva Pessoa, Maria de Nazaré Maciel Uesugi, André Salim Khayat, Jorddy Neves Cruz, José de ArimatéiaRodrigues do Rêgo, Maria Regina Sarkis Peixoto Joele, Davi do Socorro Barros Brasil

**Affiliations:** ^1^ Programa De Pós‐Graduação em Inovação Farmacêutica Universidade Federal do Pará Belém Pará Brazil; ^2^ Programa De Pós‐Graduação em Ciência e Meio Ambiente Universidade Federal do Pará Belém Pará Brazil; ^3^ Núcleo De Pesquisa em Oncologia Universidade Federal do Pará Belém Pará Brazil; ^4^ Laboratório De Mineralogia e Geoquímica Aplicada Universidade Federal do Pará Belém Pará Brazil; ^5^ Instituto De Ciências Exatas e Naturais Universidade Federal do Pará Belém Pará Brazil; ^6^ Laboratório De Cultura Celular e Efeitos Bioativos Universidade Federal do Pará Belém Pará Brazil; ^7^ Programa De Pós‐Graduação em Física Universidade Federal do Pará Belém Pará Brazil; ^8^ Programa De Pós‐Graduação em Agronomia (PGAGRO) Universidade Federal Rural Da Amazônia Belém Pará Brazil; ^9^ Instituto De Ciências Biológicas Universidade Federal do Pará Belém Pará Brazil; ^10^ Centro De Ciências Biológicas e Da Saúde Departamento De Morfofisiologia e Ciências Fisiológicas Universidade Estadual do Pará Belém Pará Brazil; ^11^ Programa De Pós‐Graduação em Ciência e Tecnologia De Alimentos Universidade Federal do Pará Belém Pará Brazil

**Keywords:** cytotoxicity, eco‐friendly, nanoparticles, piperine, silver

## Abstract

The green synthesis of silver nanoparticles (AgNPs) offers an environmentally friendly alternative to conventional methods by replacing toxic chemical reagents with natural sources. These methods utilize microorganisms, such as bacteria and fungi, or natural plant extracts rich in secondary metabolites that act as reducing and stabilizing agents during nanoparticle formation. In this study, we biosynthesized AgNPs using an ethanolic extract of *Piper nigrum* seeds and isolated piperine (PPN), the main bioactive compound. Furthermore, we evaluated the cytotoxic properties of PPN and its interaction with AgNPs (Np‐AgPPN) in gastric cancer cells. The samples were characterized using UV–visible (UV–Vis) spectroscopy, Fourier transform infrared (FTIR), scanning electron microscopy with energy‐dispersive x‐ray spectroscopy (SEM–EDS), x‐ray diffraction (XRD), thermogravimetric (TG), and differential scanning calorimetry (DSC). Results suggest successful functionalization of AgNPs with piperine (Np‐AgPPN). UV–Vis spectroscopy showed a hypsochromic shift of the maximum peak and a hypochromic effect, suggesting electronic interactions between PPN and Ag. FTIR‐attenuated total reflectance (ATR) spectra revealed shifts in the carbonyl and aromatic bands, confirming PPN–Ag complex formation. SEM micrographs displayed rectangular crystals characteristic of PPN, whereas Np‐AgPPN exhibited amorphous clusters. XRD analysis revealed a decrease in crystallinity and peaks characteristic of metallic Ag. Thermal analyses (TG/DTG and DSC) demonstrated the reduced thermal stability of Np‐AgPPN compared to PPN, in addition to altered endothermic and exothermic transitions. In vitro cytotoxicity assays showed that Np‐AgPPN had superior antitumor activity compared to PPN, with IC_50_ in the range 23.1–33.9 µg/mL, particularly against the gastric cell line AGP01 PIWIL1^−^/^−^. These results demonstrate that piperine‐functionalized AgNPs can be synthesized from a piperine‐rich extract and that Np‐AgPPN exhibits superior cytotoxic effects against gastric cancer cells. In conclusion, this work highlights the feasibility of producing piperine‐functionalized AgNPs through a green synthesis approach and demonstrates their enhanced cytotoxic activity against gastric cancer cells, reinforcing the therapeutic potential of this strategy.

## Graphical abstract

This study reports the green biosynthesis of piperine‐functionalized silver nanoparticles from Piper nigrum seed extract, followed by comprehensive physicochemical characterization and in vitro cytotoxic evaluation. The results confirm successful nanocomposite formation and demonstrate a marked enhancement of cytotoxic activity against gastric cancer cell lines, highlighting the potential of this eco‐friendly nanosystem for therapeutic applications.

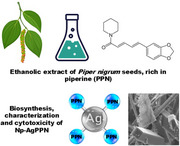

## 1 Introduction

In 1959, physicist Richard Feynman introduced the concept of nanotechnology in his seminal lecture titled “There's Plenty of Room at the Bottom” [1]. As the formal introduction of the term “nanotechnology” by Norio Taniguchi in 1974, the field has evolved rapidly, driving innovations in medicine, pharmacy, electronics, and materials science, and establishing nanotechnology as a key area of modern research [1, 2].

Among the various nanomaterials, silver nanoparticles (AgNPs) are notable for their wide applicability and unique physicochemical properties, particularly their antimicrobial activity, tunable surface chemistry, and ability to interact with organic molecules, which enables effective functionalization [3, 4]. These characteristics make AgNPs especially attractive for biomedical and pharmacological applications, including drug delivery and anticancer strategies [5].

To meet the growing demand for AgNPs, several synthesis routes have been explored, including physical, chemical, and advanced techniques. However, many of these approaches require high energy input or toxic reagents, limiting their biomedical applicability [6, 7]. In this context, green synthesis has emerged as a sustainable alternative, offering low energy consumption and reduced use of hazardous chemicals, thereby minimizing risks to human health and the environment [8, 9]. Advanced techniques, such as lithography, laser irradiation, thermal decomposition, and cryochemical synthesis, have also been developed for the controlled production of NPs [8, 10].

The biosynthesis of nanoparticles from plant extracts has emerged as a sustainable alternative to conventional production methods [11, 12]. This approach offers significant advantages, such as low energy consumption and reduced or no use of toxic solvents, thereby minimizing impact on human health and the environment [11, 13].

Medicinal plants are rich in bioactive compounds that can facilitate the biosynthesis of AgNPs. They contain alkaloids, flavonoids, and other secondary metabolites with functional groups, such as carboxylic acid (─COOH), aldehyde (─CHO), hydroxyl (─OH), and amines (─NH_2_). These groups confer redox capacity, enabling the compounds to act as reducing agents during AgNP biosynthesis. Furthermore, these compounds can facilitate the conservation and functionalization of NPs, providing them with additional properties and applications [14, 15].

To achieve this, compounds, such as extracts, essential oils, and isolates, can be obtained in various ways. The most common approach is the use of crude extracts, which contain a variety of molecules that aid in NP stabilization, whereas essential oils are rich in volatile compounds with significant reducing potential [16, 17].

In this study, we synthesized piperine‐functionalized AgNPs (Np‐AgPPN) through a biosynthetic process using *Piper nigrum* extract, a medicinal plant recognized for its high piperine (PPN) content. It is important to highlight that, prior to the functionalization process, piperine was previously isolated and purified, allowing us to specifically evaluate the role of this compound in the formation and stabilization of the nanoparticles. The NP‐AgPPN was extensively characterized using UV–visible (UV–Vis), attenuated total reflectance (ATR)‐Fourier transform infrared (FTIR), scanning electron microscopy with energy‐dispersive x‐ray spectroscopy (SEM–EDS), x‐ray diffraction (XRD), and differential scanning calorimetry (DSC). Finally, the cytotoxicity of the nanoparticles was evaluated against tumor cell lines, such as AGP‐01 (gastric ascites), AGP‐01 PIWI (−/−) (gastric ascites with inactivated PIWI gene), ACP02 (primary gastric adenocarcinoma of the diffuse type), and the nonneoplastic cell line HEK‐293 (nonneoplastic human kidney).

## 2 Materials and Methods

### 2.1 Preparation of *P. nigrum* Extract

*P. nigrum* seeds were obtained from the Águas do Caripi farm in the municipality of Igarapé‐Açu, Pará State, Brazil. The voucher specimen of the plant material was preserved and deposited in the collection of the João Murça Pires Herbarium at the Museu Paraense Emílio Goeldi. This specimen was specifically added to the Aromatic Plants of the Amazon collection in Belém, under registration number 485764.

They were dried in an air‐circulating oven (Quimis, model Q314M, Brazil) at 40°C for 72 h to stabilize the moisture content. Finally, the plant material was ground using a knife mill (Tecnal, model R‐TE‐650/1; Brazil). The extract was prepared by mixing 200 g of plant material with 1000 mL of absolute ethanol (Sigma‐Aldrich, USA). The mixture was allowed to rest on a bench for 14 days. The resulting solution was vacuum‐filtered using Whatman paper No. 1 (Sigma‐Aldrich, USA) [18, 19].

### 2.2 PPN Isolation

PPN was isolated using the green method described in a previous study [20]. In brief, the compound was isolated from an ethanolic extract of *P. nigrum* without the addition of alkalizing agents using a 1:4 mixture of extract to distilled water. The mixture was allowed to rest for 7 days to form crystalline PPN. It was then filtered through a Whatman No. 1 filter paper (Sigma‐Aldrich, USA). The retained solid was dried in an air‐circulating oven (Quimis, model Q314M, Brazil) at 40°C for 72 h.

### 2.3 Green Synthesis of Silver Nanoparticles From *P. nigrum* Extract

The green synthesis of silver nanoparticles (Np‐AgPPN) without the use of NaBH_4_ was performed using an analytical‐grade ethanolic solution (Sigma‐Aldrich, USA) containing 10 mM silver nitrate (AgNO_3_) (NEON, Brazil, batch no. 74369, purity 99.8%). This solution was slowly added to a volume of PPN‐rich *P. nigrum* extract (1:5), as described in our previous studies [18, 19], under magnetic stirring (Snijders, model 34532, Netherlands) for ∼2 h. At the end of the reaction, distilled water was added to the mixture at a ratio of 1:4 (mixture:distilled water). The mixture was allowed to rest for 7 days. Finally, it was filtered through Whatman paper No. 1, resulting in a brown crystalline solid containing AgNPs and piperine (Np‐AgPPN), the main chemical compound in this extract.

### 2.4 Colorimetric Analysis of PPN and Np‐AgPPN

Colorimetric analysis was used to evaluate the differences between the characteristic colors of PPN and NP‐AgPPN. For this evaluation, a portable colorimeter (Minolta, model CR 400, USA) was used, where the parameters L* (brightness) and b* (yellow intensity) were obtained, and the values of C* (chroma), h* (hue angle), and Δ*E* (color difference) were calculated.

### 2.5 UV–Visible Spectroscopy

UV–Vis absorption spectroscopy was used to monitor the formation of AgNPs. The optical absorption spectra of PPN and Np‐AgPPN were obtained using a UV–Vis spectrophotometer (BEL Photonics, model UV‐M51, Brazil). First, 1 mg of each sample was dispersed in 1.5 mL of ethanol and sonicated for 30 min. The spectra were recorded in the ranges 180–600 nm.

### 2.6 Attenuated Total Reflectance Fourier Transform Infrared Spectroscopy

ATR‐FTIR measurements were performed after PPN extraction and Np‐AgPPN synthesis to chemically characterize PPN and identify the organic compounds bound to Np‐AgPPN. The spectra were obtained using an absorption spectroscope (Bruker, model Vertex 70, Germany) with a spectral resolution of 4 cm^−1^. Measurements were made between 400 and 4000 cm^−1^ using ATR.

### 2.7 Scanning Electron Microscopy With Energy‐Dispersive X‐Ray Spectroscopy

SEM–EDS was used to obtain micrographs of the PPN crystals and investigate the morphology of Np‐AgPPN and the chemical compositions of the samples. The micro‐morphologies of the samples were obtained using SEM (Hitachi, model TM 3000, Japan), and the chemical compositions were obtained using EDS (Oxford Instruments, model EDS 3000, UK). The SEM–EDS analysis conditions were as follows: 15.0 vacuum: low; sample preparation: no metallization; image magnification: 15–30 000× (digital zoom 2×, 4×); image acquisition time: 30 s; and chemical analysis time: 60 s.

### 2.8 X‐Ray Diffraction

XRD was used to evaluate the crystal patterns of PPN and NP‐AgPPN. Analyses were performed using an x‐ray diffractometer (Bruker, D2 PHASER, Germany) equipped with a high‐resolution Lynxeye detector, with the voltage and current set at 30 kV and 10 mA, respectively. Cu *K*α radiation (*λ* = 1.54184 Å) was used to collect information in the angular range 4°–60° with steps of 0.02°.

### 2.9 Thermogravimetric Analysis (TG) and DSC

Simultaneous thermal analysis using TG/DSC was performed to investigate the thermal stability and transitions of the PPN and NP‐AgPPN samples. The analyses were performed in a simultaneous thermal analyzer (Netzsch, model STA 449 F5 Jupiter, Germany) equipped with a vertical cylindrical furnace and N_2_ flow of 50 mL/s at a heating rate of 10°C/min and temperature range of 30–1100°C.

### 2.10 In Vitro Cytotoxicity Activity

#### 2.10.1 Cell Culture

The tumor cell lines employed in this study were as follows: Paraense gastric adenocarcinoma 01 (AGP‐01, gastric ascites), Paraense gastric adenocarcinoma 01 *PIWIL1* knockout (AGP‐01 PIWI (−/−), gastric ascites with *PIWIL1* gene inactivation), and Paraense adenocarcinoma 02 [21] (ACP02, primary diffuse‐type gastric adenocarcinoma) [22] were established at the Oncology Research Center of the Federal University of Pará and exhibit a phenotypic profile characteristic of the Brazilian Amazon region. The nonneoplastic HEK‐293 cell line (human embryonic kidney), obtained as a donation from the Cell Bank of the Evandro Chagas Institute, was included for comparative analyses. Cells were cultured in DMEM medium supplemented with 10% fetal bovine serum and 1% antibiotics (penicillin/streptomycin) and maintained in an incubator at 37°C under a humidified atmosphere containing 5% CO_2_.

#### 2.10.2 Cytotoxicity Assay

Investigating the cell viability and cytotoxicity of PPN and Np‐AgPPN is essential for assessing their safety and therapeutic potential. The MTT assay is based on the reduction of 3‐(4,5‐dimethyl‐2‐thiazol)‐2,5‐diphenyl‐2‐*H*‐tetrazolium bromide, a yellow tetrazolium dye, to purple formazan crystals from mitochondrial dehydrogenase substrates that are present only in metabolically active cells. The formazan product obtained was then analyzed spectrophotometrically, and the spectra of the treated and untreated cells were used to estimate the extent of cytotoxicity [23]. Cells were seeded in 96‐well plates at a density of 3 × 103 cells per well. PPN and Np‐AgPPN were dissolved in dimethyl sulfoxide (DMSO) to obtain an initial concentration of 10 mg/mL and were added to a 96‐well plate (100 µL/well). A dose‐response assay was performed with a concentration curve ranging from 100 to 1.56 µg/mL for a 72‐h treatment period in a 5% CO_2_ incubator. Subsequently, the plates were removed, the supernatant was aspirated, and 100 µL of a 0.5 mg/mL MTT solution diluted in DMEM was added. The samples were then incubated again in the 5% CO_2_ incubator for 3 h. The supernatant was subsequently aspirated, and the precipitate was resuspended in 100 µL of DMSO and shaken for 10 min until the formazan crystals were completely dissolved. The plates were analyzed using a microplate spectrophotometer at a wavelength of 570 nm.

#### 2.10.3 Data Analysis

The effects of PPN and Np‐AgPNN on cell viability were obtained from the percentages relative to the negative control using Equation (1), where Absexp and Absctr account for the absorbance at 570 nm for the experimental and control samples, respectively. After verifying data normality using the Shapiro–Wilk test, differences between groups were analyzed using two‐way ANOVA followed by Bonferroni post hoc testing, with significance levels of *p* > 0.005 (***) and *p* > 0.0001 (***) considered statistically significant.

(1) $$\mathrm{Cell}\;\mathrm{viability}\;\left(\%\right)=\left(\frac{\mathrm{Ab}s_{\exp}}{\mathrm{Ab}s_{\mathrm{ctr}}}\right)\times 100$$

Subsequently, the average concentrations of PPN and Np‐AgPNN capable of causing 50% of the maximum effect (CI_50_) and their respective confidence intervals (CI95%) for each lineage were obtained from the nonlinear regression in GraphPad Prism version 8.0.

## 3 Results and Discussions

The recent literature presents several studies involving the green synthesis of silver nanoparticles; however, the system developed in this work exhibits distinctive characteristics when compared with these models. Shumi et al. [24] reported the production of AgNPs mediated by *Lippia abyssinica* extract, subsequently functionalized with histidine and phenylalanine. Their nanoparticles exhibited antibacterial activity and antioxidant action, with characterizations based on UV–Vis, FTIR, XRD, and electron microscopy.

Similarly, Korpayev et al. [12] synthesized AgNPs using floral extracts of *Alhagi persarum*, identifying flavonoids and phenolic acids as the main reducing agents. In addition to the antioxidant and antimicrobial profiles, these AgNPs demonstrated cytotoxicity toward the MCF‐7 cell line and were characterized by UV–Vis, FTIR, XRD, SEM/TEM, and EDS analyses.

Another relevant study is that of Demarchi et al. [25], in which AgNPs were incorporated into a magnetic *O*‐carboxymethyl chitosan/γ‐Fe_2_O_3_ nanocomposite reduced by *Eugenia umbelliflora* extract, resulting in a system with significant antimicrobial activity and toxicity evaluation in *Artemia salina* and *Cucumis sativus*.

In contrast to these studies, which employ complex plant extracts or amino acids as reducing/stabilizing agents, the present study uses piperine previously isolated and purified as the main functionalizing molecule, allowing a more direct relationship between the structure of the bioactive compound and the physicochemical properties of the Np‐AgPPN. Moreover, although the aforementioned studies mainly explore antimicrobial activities or cytotoxicity in a limited set of tumor cell lines, the present work comprehensively evaluates the cytotoxic behavior of Np‐AgPPN in multiple human gastric cancer cell lines (AGP‐01, AGP‐01 PIWIL1^−^/^−^, and ACP02), in addition to a normal cell line (HEK‐293). Thus, the results presented here expand current knowledge by demonstrating the selective cytotoxic potential of nanoparticles functionalized exclusively with piperine, which distinguishes this system from previously described models in the recent literature.

### 3.1 Color Analysis

This analysis was performed to evaluate the color change in the filtered material resulting from the formation of PPN‐functionalized nanoparticles. To this end, isolated PPN and Np‐AgPPN were placed on a flat surface and analyzed using a portable colorimeter.

**FIGURE 1:**
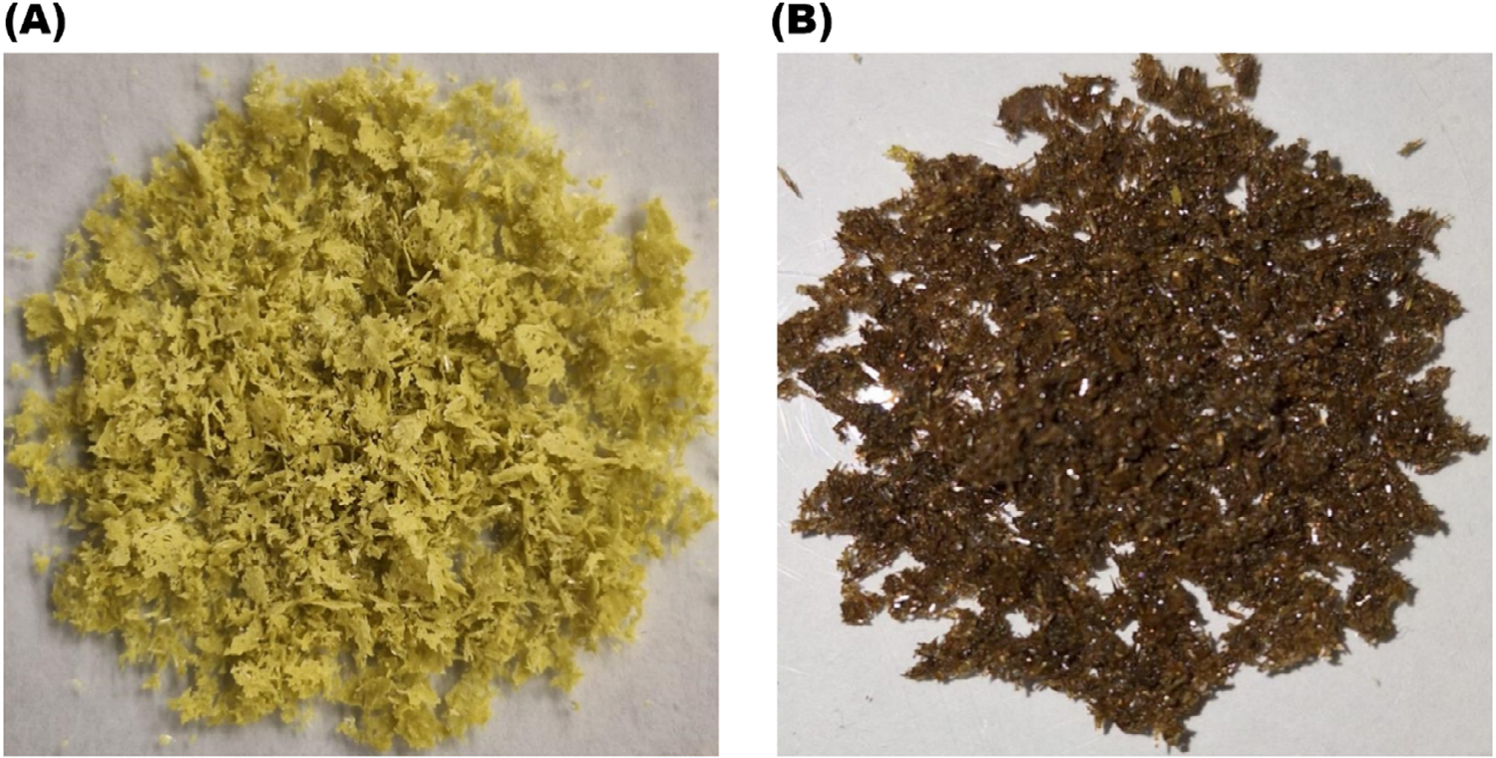
Comparison of (A) crystalline solid PPN with (B) Np‐AgPPN.

In Figure 1, we observe striking visual differences; PPN exhibits a greenish‐yellow color, whereas Np‐AgPPN displays a dark brown hue. Considering the colors observed, the formation of Np‐AgPPN from the PPN‐rich *P. nigrum* extract caused noticeable visual changes, with the color change being a strong indication of nanoparticle formation, according to Shumi et al. [24].

This color change is a classical indication of the formation of metallic nanoparticles and is associated with the phenomenon of surface plasmon resonance (SPR). SPR occurs due to the collective oscillation of conduction electrons on the surface of silver nanoparticles when excited by light, resulting in characteristic absorption bands in the UV–Vis region and in the typical dark‐brown coloration of these nanoparticles. This optical behavior has been widely reported for AgNPs obtained through green synthesis using plant extracts [12, 24].

To quantify the visual color distinctions, we used a colorimeter to evaluate the lightness, color tendency, chroma, and hue angle of the samples. The results are presented in Table 1.

**TABLE 1:** Color parameter results for the PPN and Np‐AgPPN samples.

Parameters	PPN	Np‐AgPPN
L*	37.92 ± 0.48	17.26 ± 0.10
b*	+19.57 ± 0.14	+2.51 ± 0.35
C*	19.57 ± 0.14	3.15 ± 0.31
Hue	90.81 ± 0.52	59.24 ± 2.65
Δ*E*	—	26.87 ± 0.54

*Note*: Here L* is the lightness parameter, b* is the color component on the blue–yellow axis, C* is the chroma, hue is the hue angle, and Δ*E* is the total color difference between the two samples.

Luminosity (L*) is a parameter that ranges from 0 to 100, where values close to 0 indicate that the sample tends toward black, whereas values close to 100 represent lighter colors or colors close to white. Our results showed that PPN had a value of L* = 37.92 ± 0.48, whereas Np‐AgPPN had L* = 17.26 ± 0.10, indicating that the sample with silver nanoparticles was significantly darker.

The parameter b* represents the color direction along the blue–yellow axis. Positive values indicate yellow hues, and negative values indicate blue hues. PPN had b* = +19.57 ± 0.14, revealing a strong tendency toward yellow, whereas Np‐AgPPN had b* = + 2.51 ± 0.35, indicating that the yellowish intensity was significantly reduced in the presence of silver nanoparticles. Chroma (C*), or color saturation, represents the intensity of the perceived hue, with higher values indicating more vivid and saturated colors and lower values indicating dull or grayish colors. PPN had C* = 19.57 ± 0.14, indicating an intense and saturated color, whereas Np‐AgPPN presented C* = 3.15 ± 0.31, indicating a significant reduction in color intensity.

The hue angle (hue) expresses the dominant hue of a color, ranging from 0° to 360°, where 0° corresponds to red, 90° to yellow, 180° to green, and 270° to blue. The PPN sample had a hue of 90.81° ± 0.52°, indicating a predominantly yellow hue. The Np‐AgPPN had a hue of 59.24° ± 2.65°, indicating a shift toward shades closer to orange, suggesting a perceptible color change.

The Δ*E* parameter represents the total color difference between the two samples, with values greater than 3 being perceptible to the naked eye. The Δ*E* value between PPN and Np‐AgPPN was 26.87 ± 0.54, indicating a marked visual difference and confirming that the incorporation of silver nanoparticles significantly altered the appearance of the sample.

The results revealed a significant difference (*p* ≤ 0.05) in luminosity (L*), with PPN presenting greater luminosity and maintaining its yellow color. This result suggests good structural and color stability, as observed by Ramos et al. [20].

The b* parameter, which indicates a tendency toward yellow coloration when it presents positive values, also showed a significant difference between PPN and Np‐AgPPN (*p* ≤ 0.05). The PPN sample maintained its yellow hue, whereas Np‐AgPPN showed a noticeable change to dark brown, both at time zero and after 7 days of rest. These results are consistent with the findings of Pathak et al. [26], who observed a color change from clear to dark red and, later, dark brown during the formation of silver nanoparticles (AgNPs) with extracts of *Scytonema geitleri* HKAR‐12 and silver nitrate (AgNO_3_).

Furthermore, PPN showed significantly higher (*p* ≤ 0.05) chroma (C*) and hue angle (Hue) values compared to Np‐AgPPN, indicating a transition from yellow to deep‐brown (Table 1). Chroma is associated with color saturation or intensity, whereas the hue angle refers to the hue or perceived color [27]. The Np‐AgPPN sample exhibited greater color intensity, suggesting that the presence of silver nitrate (AgNO_3_) induced significant chromatic changes during crystal formation.

The influence of silver on the color of the formed solid had a direct impact on the visual distinction of piperine, as demonstrated by the analyzed parameters. The images of PPN and Np‐AgPPN presented in Table 1 reveal that PPN resembles piperine samples characterized as crystalline solids by Alves et al. [19] and Sulman [28]. The change in the color of Np‐AgPPN from yellow to dark brown suggests the formation of piperine crystals containing silver, indicating the presence of silver nanoparticles, as described by Pathak et al. [26].

Thus, the colorimetric data in Table 1 corroborate the visual observations showing changes in the color of PPN and Np‐AgPPN. These changes were quantified using parameters such as L*, C*, Hue, and Δ*E*, evidencing a color transition from greenish‐yellow (PPN) to dark brown (Np‐AgPPN).

### 3.2 UV–Visible Spectroscopy

**FIGURE 2:**
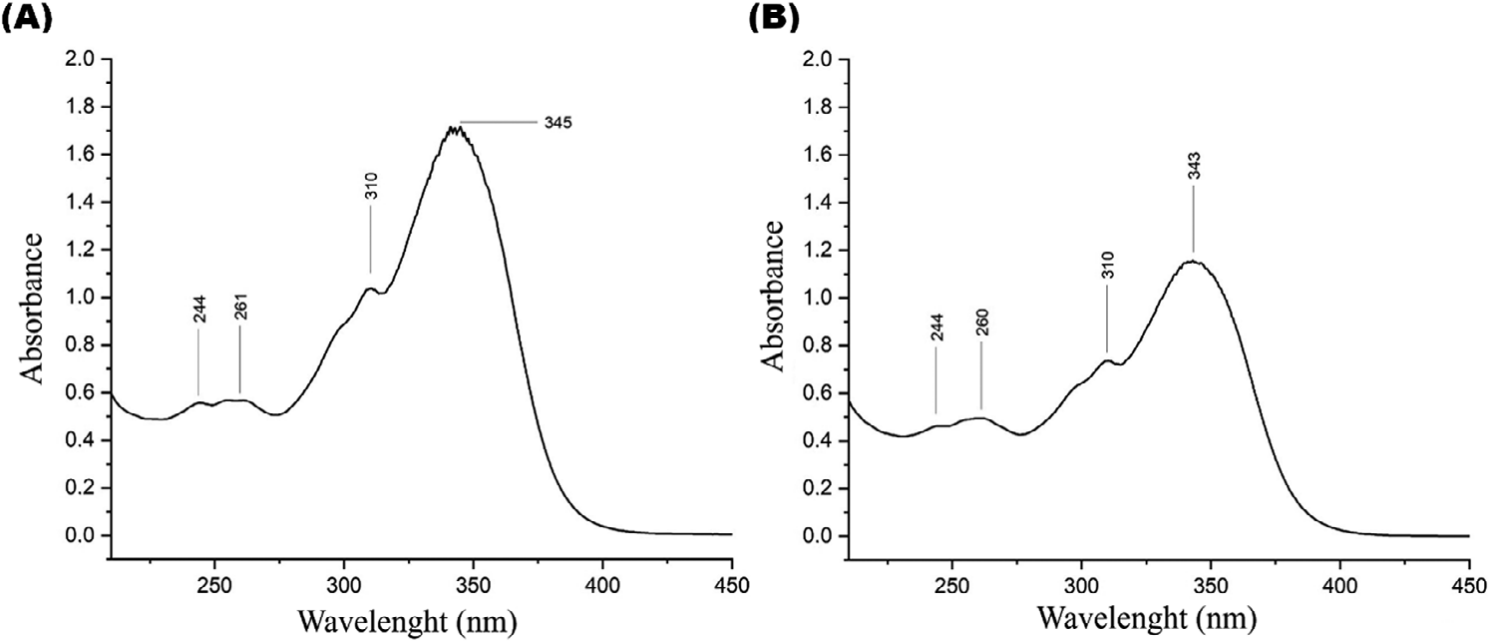
UV–Vis absorption spectra of (A) isolated piperine (PPN) and (B) piperine‐functionalized silver nanoparticles (Np‐AgPPN). The analyses were performed using a BEL photonics UV–Vis spectrophotometer, model UV‐M51 (Brazil). For each measurement, 1 mg of sample was dispersed in 1.5 mL of ethanol and subjected to sonication for 30 min. Spectra were acquired in the 180–600 nm range at room temperature to monitor the formation of silver nanoparticles and to evaluate spectral variations associated with π → π and *n* → π electronic transitions. A decrease in absorbance intensity and a hypsochromic shift of the main band are observed in the Np‐AgPPN sample, indicating electronic reorganization resulting from coordination between PPN and Ag^+^.

UV–Vis spectroscopy was used to confirm the formation of PPN‐functionalized AgNPs by detecting changes in the absorbance profiles of the isolated PPN and Np‐AgPPN samples after PPN functionalization. The samples were analyzed in the 200–450 nm range with a spectral resolution of 1 nm. The obtained spectra were compared to identify characteristic absorption peaks and evaluate changes in the intensity and maximum absorbance wavelength related to nanoparticle formation and the interactions between PPN and silver ions. The results of the spectrophotometric analyses are shown in Figure 2.

The spectrophotometric analyses showed absorption variations between 244 and 350 nm, which is the characteristic range of π → π* and *n* → π* electronic transitions in organic molecules containing conjugated systems. The peaks around 350 nm were well‐defined but exhibited different intensities.

The PPN spectrum exhibited absorption bands at 244, 261, 310, and 345 nm, with the latter being the most intense (1.75). These bands are characteristic of π → π* and *n* → π* electronic transitions, which are typical of conjugated aromatic systems and carbonyl groups present in the piperine structure. Previous studies have reported that piperine exhibits maximum absorption in the 342–345 nm range.

The Np‐AgPPN spectrum exhibits a similar spectral profile, with bands at 244, 260, 310, and 343 nm. However, a significant reduction in the absorption intensity and a slight shift in the main peak from 345 to 343 nm were observed.

These changes demonstrate hypochromic spectroscopic effects, corresponding to a reduction in the absorption intensity and a hypochromic effect, characterized by a shift in the absorption band to a shorter wavelength. These effects indicate that the complexation of piperine with silver promoted electronic reorganization in the molecule, possibly through the coordination of Ag⁺ to the amide carbonyl group. This interaction reduces the conjugation of the π system, hindering electronic transitions and altering the electron density of the molecule. In contrast, bathochromic and hyperchromic effects were not observed, reinforcing the idea that there was no increase in the electronic conjugation or density of excitable states in the system.

These observations corroborate the structural modification of piperine after complexation with Ag, which may influence its optical and reactive properties; these are relevant factors for applications in bioactive and photosensitive materials.

### 3.3 Fourier Transform Infrared‐Attenuated Total Reflectance

**FIGURE 3:**
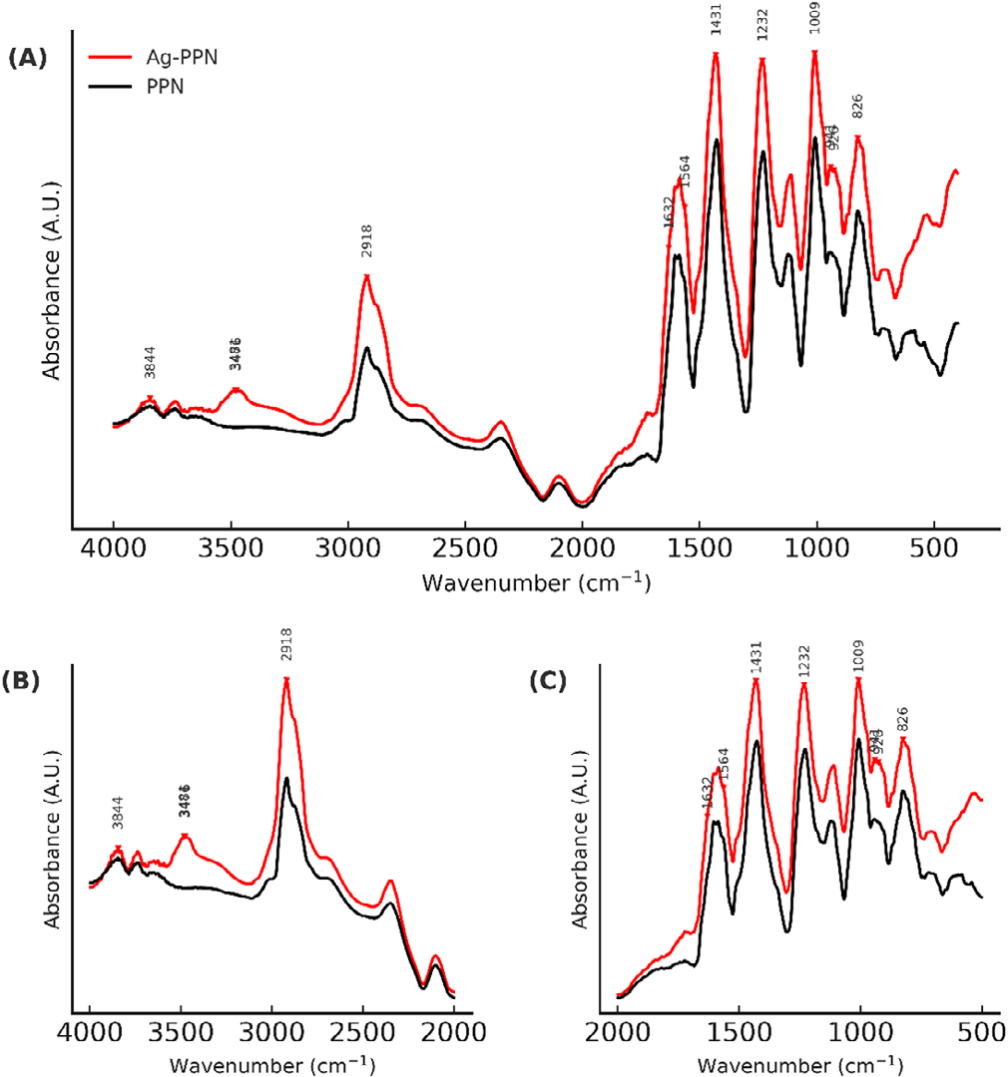
FTIR‐ATR of isolated piperine (PPN) and piperine‐functionalized silver nanoparticles (Np‐AgPPN). Analyses were carried out using a Bruker Vertex 70 spectrometer (Germany) with a spectral resolution of 4 cm^−1^ over the 400–4000 cm^−1^ range after extraction and synthesis procedures. (A) Full spectra; (B) enlarged view of the C─H stretching region (4000–2000 cm^−1^); and (C) enlarged view of the region associated with aromatic vibrations, carbonyl (C═O), and C─O─C stretching modes (2000–1000 cm^−1^). Band shifts and intensity variations indicate structural changes related to the interaction between piperine and silver.

FTIR‐ATR spectroscopy was used to identify the functional groups of the compounds and to evaluate their molecular interactions in the crystalline solids of PPN and NP‐AgPPNs. This technique is particularly useful for confirming complex formation or chemical changes induced by metallic agents, such as silver. Spectra were recorded in the range 4000–400 cm^−1^, with a resolution of 4 cm^−1^ and 32 scans per spectrum. The obtained spectra are presented in Figure 3.

In the full spectrum (Figure 3A), both samples exhibited characteristic bands in the range 4000–500 cm^−1^, with significant differences in the intensity and positioning of some of the main bands. Figure 3B,C shows the differences between the spectra of PPN and Np‐AgPPN, respectively.

The spectra revealed peaks with higher intensities for PPN than those for Np‐AgPPN. The samples presented characteristic vibrational modes located at 1033 cm^−1^ (symmetrical stretching C─O─C), 1132 cm^−1^ (asymmetrical stretching C─O─C), and 1580 cm^−1^ (aromatic stretching of the benzene ring), according to Gorgani et al. [29] and Lim et al. [30]. These signals correspond to the aromatic portion of the piperine structure [20]. For Np‐AgPPN, a shift to a lower wavenumber was observed at 1565 cm^−1^, suggesting a change in the interaction of the aromatic ring.

The amide portion of the molecule is related to the vibrational mode at 1631 cm^−1^ (C═O─N bond), as described by Kusumorini et al. [31]. The high‐energy vibrational mode centered at 2940 cm^−1^, associated with the C─H stretching in the aliphatic chain, shifted to 2920 cm^−1^ in Np‐AgPPN, indicating that less energy was required for the molecular vibration, possibly due to the interaction of the functional group with silver [32].

Peaks similar to those observed in this study have been reported by Ramos et al. [20]. According to Pereira et al. [33], changes in the amplitude and wavenumber shifts in the FTIR spectra are indicative of vibrational interactions among the components analyzed.

Additionally, a peak in the 3400–3500 cm^−1^ region was observed exclusively for the Np‐AgPPN sample. According to Kucuk et al. [34], such a signal suggests that the formation of a complex with Ag introduces new vibrational characteristics. Xu et al. [35] attributed a broad region in the range 1280–1510 cm^−1^ to the asymmetric stretching vibration of the NO_3_^−^ ion, observed in silver composites, which may corroborate the findings of the present study.

Alves et al. [19] also reported intense bands at 1635 cm^−1^ attributed to the aromatic and aliphatic stretching vibration modes of the ─C═C─ bonds, as well as 1585 cm^−1^ (asymmetric stretching of ─O═C─N─), in addition to scissor and torsion frequencies at 1490 and 1437 cm^−1^, respectively, associated with the ─CH_2_─ bonds. Other reported vibration modes were 1253 cm^−1^ (C─O─C stretching of the phenylmethylenedioxy ring) and 995 cm^−1^ (wagging motion of the ─CH_2_ group of the piperidine).

In general, the FTIR‐ATR spectra of the PPN and Np‐AgPPN samples show differences in both the peak intensity (amplitude) and wavenumber shifts, indicating structural interactions with AgNPs. Although both share the characteristic signs of PPN, the changes observed in Np‐AgPPN suggest the formation of a chemical or physical complex with Ag.

In addition to the spectroscopic data presented, the interaction between piperine and silver can be understood through a mechanistic model based on the coordination of the Ag⁺ ion with electronically rich groups of the molecule. Piperine contains a conjugated carbonyl group and an aromatic system with high electron density, both capable of acting as coordination sites. Thus, it is proposed that the oxygen atom of the carbonyl group (C═O) establishes an Ag–O type interaction, accompanied by possible secondary interactions between the metal cation and the π‐system of the aromatic ring. This dual electronic contribution favors the stabilization of the PPN–Ag complex, resulting in the band shifts observed in the FTIR spectrum, particularly in the carbonyl and aromatic regions.

### 3.4 Scanning Electron Microscopy With Energy‐Dispersive X‐Ray Spectroscopy

**FIGURE 4:**
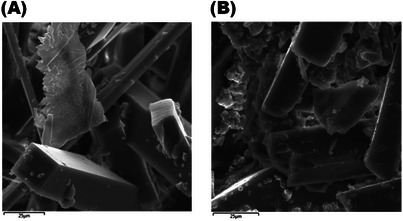
SEM micrographs of (A) isolated piperine (PPN) and (B) piperine‐functionalized silver nanoparticles (Np‐AgPPN), acquired at a magnification corresponding to 25 µm. The images reveal the regular crystalline morphology of PPN and the presence of granular aggregates and amorphous structures in Np‐AgPPN, suggesting the incorporation of silver into the organic matrix. Complementary energy‐dispersive x‐ray spectroscopy (EDS) analysis was employed for the qualitative and semiquantitative identification of chemical elements, including carbon (C), oxygen (O), nitrogen (N), and silver (Ag).

SEM analyses were performed to evaluate the surface morphologies and crystal shapes of PPN and Np‐AgPPN samples. EDS complements structural analysis by identifying the qualitative and semiquantitative chemical compositions of compounds. The morphologies of the samples were observed by obtaining images at 25 µm magnification, where the presences of the elements silver (Ag), carbon (C), oxygen (O), and nitrogen (N) were detected. The micrographs are shown in Figure 4 and the elemental compositions are listed in Table 2.

**TABLE 2:** Mass composition (wt%) of the chemical elements in the PPN and Np‐AgPPN samples.

Sample	Weight %			
	C	O	N	Ag
PPN	72.8	15.6	11.2	—
Np‐AgPPN	63.2	22	12.7	2.1

Figure 4A shows piperine crystals with a well‐defined regular morphology and an elongated shape, which are characteristic of piperine [19]. The PPN crystals were well‐formed, predominantly rectangular with a cubic face, in the form of rods with variable micrometric dimensions and smooth, thick surfaces. Rectangular crystals with a cubic face observed in PPN were characterized as monoclinic by Ramos et al. [20] and Alves et al. [19].

In Figure 4B, even with the interaction of Ag with PPN to form Np‐AgPPN, the PPN structure is still visible, but with the presence of amorphous clusters, suggesting the interaction of PPN with Ag. The presence of amorphous clusters in Np‐AgPPN corroborates the findings of Pathak et al. [26] and Demarchi et al. [25], who reported that nanoscale metal particles tend to form amorphous structures.

The irregular crystal growth observed in Np‐AgPPN may also be related to the interactions between the crystal surface and the solvent because organic solvents reduce interfacial tension, promoting the transition from smooth to rough surfaces and accelerating structural growth. The presence of Ag in the solution significantly influenced these changes, as discussed by Padalkar and Gaikar [36].

Semiquantitative elemental analysis by EDS showed composition differences between Np‐AgPPN and PPN. According to the results presented in Table 2, PPN contains carbon, oxygen, and nitrogen contents of 72.8%, 15.6%, and 11.2%, respectively, which agrees with the results presented by Ramos et al. [20].

The Np‐AgPPN sample showed a reduction in the carbon content to 63.2% and an increase in the oxygen (22.0%) and nitrogen (12.7%) contents, in addition to a 2.1% increase in silver. The decrease in carbon content, together with the increase in oxygen and nitrogen contents, may be related to the interaction of Ag with O and N, which favors the interaction of the metallic element. This process shows that the PPN structure can serve as a basis for Ag anchoring in the formation of Np‐AgPPN, similar to the process described by Thang et al. [34], who studied the interaction of sulfur‐containing amino acids in HHK as anchoring points for AgNPs.

### 3.5 Particle Size Distribution and Zeta Potential Analysis

**FIGURE 5:**
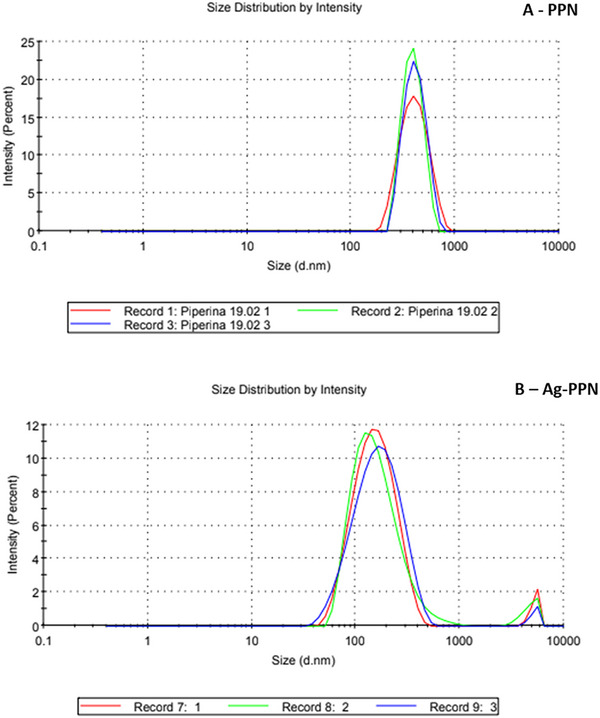
Intensity‐weighted hydrodynamic size distribution obtained by dynamic light scattering (DLS) for (A) isolated piperine (PPN) and (B) piperine‐functionalized silver nanoparticles (Np‐AgPPN). Analyses were performed at 25°C, with samples previously dispersed in an appropriate solvent and subjected to sonication to ensure adequate colloidal dispersion. Each curve represents independent measurements (*n* = 3). PPN exhibited a relatively narrow unimodal distribution, whereas Np‐AgPPN displayed a main peak in the nanoscale range (∼120–180 nm) and a secondary micrometric population associated with aggregate formation, a behavior typical of metallic nanoparticles stabilized by organic ligands.

Although SEM images acquired at a magnification of 25 µm provided relevant information on the overall morphology and surface features of the samples, complementary analyses were required to confirm nanoscale dimensions and the colloidal behavior of the system. Therefore, dynamic light scattering (DLS) and zeta potential (*ζ*) analyses were performed to investigate the hydrodynamic size distribution and surface charge of PPN and Np‐AgPPN (Figure 5).

The zeta potential (*ζ*) behavior observed for isolated piperine (PPN) and piperine‐functionalized silver nanoparticles (Np‐AgPPN) is fully consistent with data reported in the literature and with the expected mechanism of nanocomposite formation (Figure 5). For the PPN system, moderately negative *ζ* values (−18 to −25 mV) indicate predominantly electrostatic colloidal stability, characteristic of piperine‐based systems, a finding corroborated by the relatively narrow hydrodynamic size distribution observed in the DLS analyses.

Upon silver incorporation, a reduction in the magnitude of *ζ* values to the −10 to −15 mV range was observed. This effect is attributed to coordination between silver (Ag⁺/Ag^0^) species and the functional groups of piperine, as well as to surface reorganization of the particles, resulting in a decreased density of available ionizable groups. This surface modification explains both the shift of the main size distribution peak toward smaller hydrodynamic dimensions (∼120–180 nm) and the emergence of a secondary micrometric population associated with aggregate formation, as evidenced by the intensity‐based DLS distribution (Figure 5).

In this context, the reduction in zeta potential for the Np‐AgPPN system should not be interpreted as an indicator of critical colloidal instability, but rather as evidence of a transition in the stabilization mechanism, shifting from predominantly electrostatic to primarily steric. This steric stabilization is provided by the organic piperine layer adsorbed or coordinated onto the surface of the silver nanoparticles, which acts as a physical barrier against excessive aggregation. Accordingly, the differences observed in *ζ* values between the PPN and Np‐AgPPN systems are expected, coherent with the hydrodynamic distribution profiles obtained by DLS, and fully compatible with the proposed model for the formation of the piperine–silver nanocomposite.

### 3.6 X‐Ray Diffraction

**FIGURE 6:**
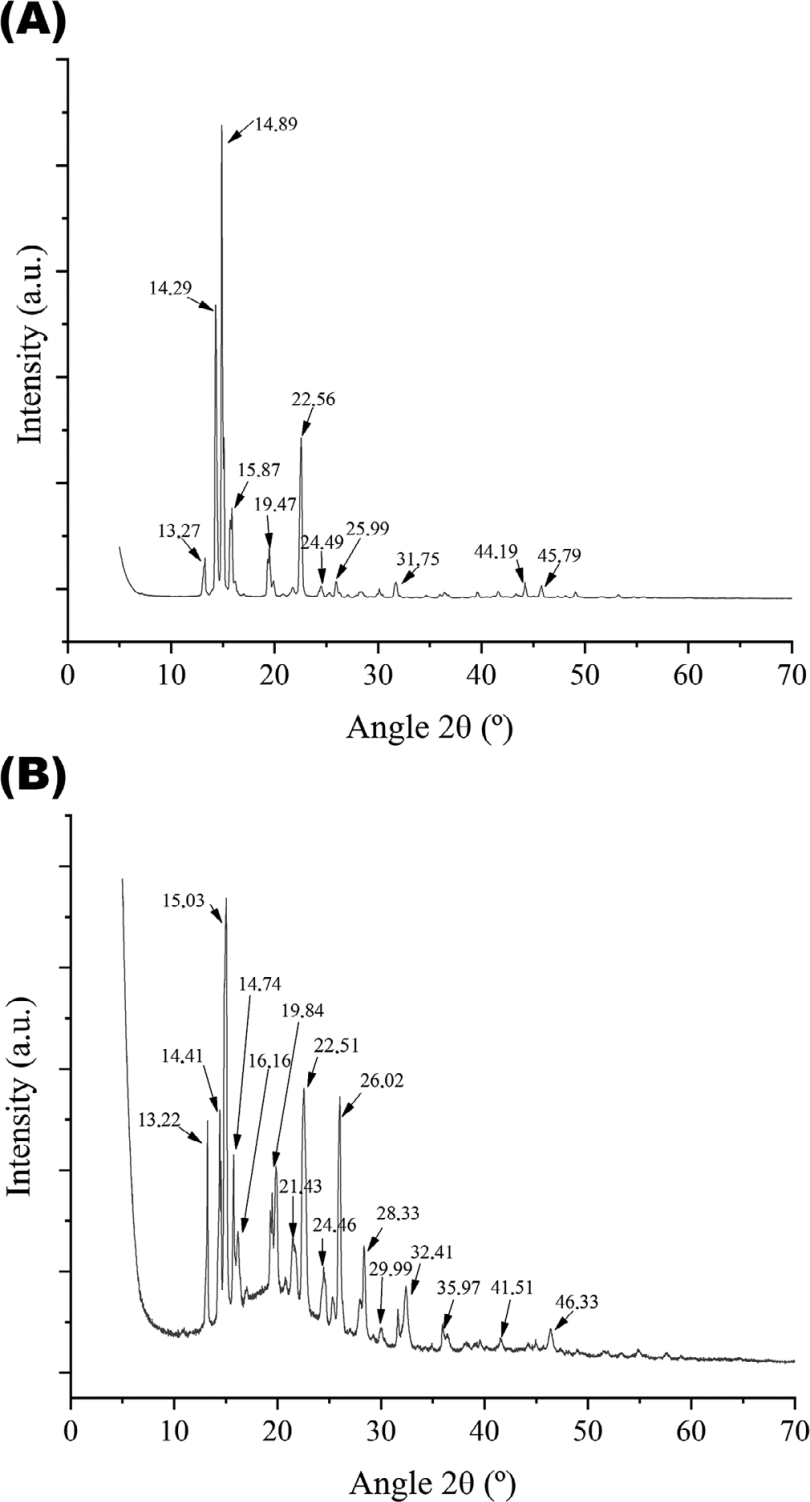
Diffractograms of (A) NNP and (B) Np‐AgPPN.

XRD analyses were performed to evaluate the structural characteristics and degree of crystallinity of the PPN and NP‐AgPPN samples. For this purpose, the characteristic diffraction patterns of PPN were obtained, in which we also detected structural modifications caused by the interaction of PPN with Ag after the formation of the nanoparticles. The obtained diffractograms are presented in Figure 6.

The diffractograms obtained revealed well‐defined, high‐intensity peaks for NNP in the regions: 2*θ* = 14.29° (*d* = 6.20 Å; *I*% = 63.59), 2*θ* = 14.87° (*d* = 5.96 Å; *I*% = 100), and 2*θ* = 22.56° (*d* = 3.94 Å; *I*% = 35.89). Furthermore, medium intensity peaks were observed at 2*θ* = 13.27° (*d* = 6.67 Å; *I*% = 8.63), 2*θ* = 15.87° (*d* = 5.58 Å; *I*% = 20.16), and 2*θ* = 19.47° (*d* = 4.56 Å; *I*% = 11.05). Additional lower intensity peaks between 2*θ* = 24.49° and 50° confirmed the crystalline nature of the compound, as reported by Ramos et al. The literature describes that, when analyzing piperine using XRD, reflections can occur in a wide range of 2*θ* angles (4°–60°) [37, 38, 39].

More intense peaks were observed in the diffractogram of Np‐AgPPN. The characteristic peaks occurred at 2*θ* = 13.22° (*d* = 6.70 Å; *I*% = 50.62), 2*θ* = 14.41° (*d* = 6.15 Å; *I*% = 53.47), 2*θ* = 15.03° (*d* = 5.89 Å; *I*% = 100), 2*θ* = 22.51° (*d* = 3.94 Å; *I*% = 59.19), and 2*θ* = 26.02° (*d* = 3.42 Å; *I*% = 57.05). Additional peaks of lower intensity were observed at 2*θ* = 14.74°, 16.16°, 19.84°, 21.43°, 24.46°, 28.33°, and 32.41°. In addition, four peaks between 20° and 80° in 2*θ* (32.58°, 46.56°, 55.12°, and 76.96°) correspond to the typical pattern of Ag silver nanoparticles, indicating their crystalline nature [26].

In the diffractograms of PPN and Np‐AgPPN, characteristic diffraction peaks were also observed in the crystal structure of PPN, located at 14.89°, 15.11°, 19.47°, and 22.56° for Np‐AgPPN, and at 14.88°, 14.98°, 15.03°, and 22.54° for the sample with silver [40], which corroborates both the preservation of the PPN crystalline structure and the formation of Np‐AgPPNs. Even though the PPN diffractogram shows the emergence of a new peak and the displacement of peaks characteristic of piperine, it also suggests the formation of crystalline hydrates [29].

For the Np‐AgPPN sample, broad peaks at 2*θ* of approximately 38.1°, 44.3°, 64.4°, and 77.4°, which are associated with metallic silver with a face‐centered cubic (FCC) structure, were not detected. The absence of these signals, together with the changes in the diffraction pattern, reinforced the hypothesis of nanoparticle formation associated with piperine and silver, a phenomenon already documented for piperine with other metallic elements [41].

In the study by Nguyen et al. [42], the Gel0.1CurAg complex exhibited well‐defined diffraction peaks at 2*θ* = 38.1°, 44.3°, and 64.4°, assigned to the (1 1 1), (2 0 0), and (2 2 0) planes of metallic silver with a FCC structure, according to JCPDS card 03‐0921. These results indicate the formation of Ag^0^ nanoparticles with sufficiently developed crystalline domains to generate detectable XRD signals. In contrast, these characteristic peaks were not observed in the Np‐AgPPN composite, as the regions around 38°, 44°, and 64° did not display reflections compatible with the metallic silver phase. The only signal detected above 40° (46.33°) showed low intensity and pronounced peak broadening, which does not correspond to the typical diffraction pattern of Ag^0^.

This discrepancy relative to the behavior reported by Nguyen et al. [42] reflects fundamental structural differences between the analyzed systems. In Np‐AgPPN, silver is present as nanoparticles with an extremely reduced crystallite size, strongly stabilized and coated by the piperine organic matrix. This configuration restricts the growth of metallic domains and promotes significant peak broadening, often below the XRD detection limit. The diffraction pattern of PPN, which is intrinsically highly crystalline in the 10°–30° region, is still observed after composite formation, albeit with reduced intensity and increased broadening, indicating structural interactions between piperine and silver. Therefore, the absence of metallic Ag^0^ peaks does not indicate the absence of silver but rather results from high dispersion, extensive organic coating, and the nanometric size of the particles—conditions that favor semicrystalline or partially amorphous states. Similar behavior has also been reported for microgel–Ag systems by Nguyen et al. [42].

The peaks attributed to Np‐AgPPN may be the result of the crystallization of bioorganic phases present on the surface of the metal nanoparticles, as described by Pathak et al. [26]. These findings are consistent with the FTIR results (Figure 4), indicating an interaction between piperine and silver.

The crystallinity index (ICr%) was used to quantify the ratio of crystalline to amorphous regions in the samples. Therefore, the analyses were performed in the scanning region of 2*θ* = 5° and 60°, where the most representative peaks of both the samples could be located. The obtained data are summarized in Table 3.

**TABLE 3:** Crystallinity indices of PPN and Np‐AgPPN.

Sample	Maximum intensity	Amorphous intensity	ICr (%) Hulleman equation
PPN	8.96	4.8	53.52
Np‐AgPPN	9.37	3.03	32.34
	Crystalline area	Total area^a^	ICr (%)^b^
PPN	9.37E + 32	1.01E + 33	92.31
Np‐AgPPN	4.07E + 32	5.90E + 32	68.95

^a^ Amorphous area + crystalline area.

^b^ Crystallinity index related to amorphous diffraction areas (Integration).

The relative crystallinity index values presented in Table 3 indicate the influence of PPN content and its interaction with Ag on the structural properties of the solids. The PPN sample presented the highest ICr% values, with a smaller amorphous area, particularly between 2*θ* = 13° and 16.6°, demonstrating a higher degree of crystallinity. Despite the reduction in the relative crystallinity of the Np‐AgPPN sample, it can still be considered a crystalline material, suggesting that the presence of Ag did not compromise the structural organization but rather promoted the modification of the crystalline structure.

The XRD results demonstrated that PPN had a high degree of crystallinity, with intense peaks in regions characteristic of this compound. The formation of Np‐AgPPN altered the diffraction pattern, as evidenced by a shift in the XRD peaks owing to the presence of Ag in the compound. There was a reduction in the ICr% of Np‐AgPPN compared to that of PPN, suggesting a partial change in the structural organization; however, a crystalline structural pattern typical of a hybrid system formed by organic and inorganic compounds was observed.

### 3.7 Thermogravimetry/Derivative Thermogravimetry (TG/DTG)

**FIGURE 7:**
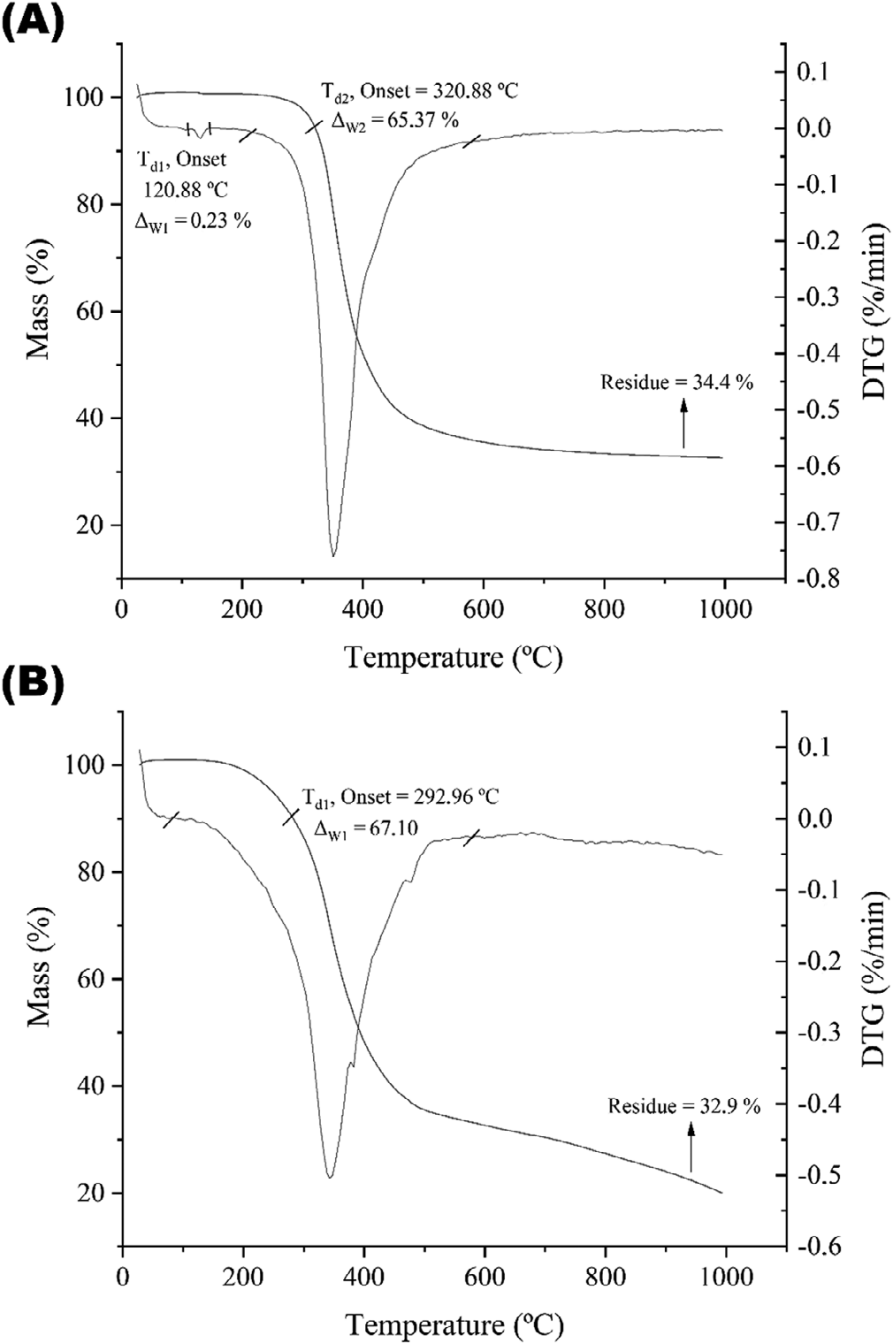
TG and DTG thermograms of (A) PPN and (B) Np‐AgPPN samples.

TG/DTG analysis was used to evaluate the thermal behavior and mass variation of the PPN and Np‐AgPPN samples as a function of temperature. This approach is important for verifying the influence of the functionalization of Ag with PPN on its physicochemical characteristics. The thermal behavior of the material was evaluated based on the curves that show the relationship between mass variation and temperature. The obtained graphs are presented in Figure 7.

The thermograms obtained by TG analysis and its derivative (DTG), shown in Figure 6, describe the thermal behavior of PPN and Np‐AgPPN, highlighting the thermal degradation events and respective mass losses (∆*w*) as a function of temperature. These data enabled inference of various aspects, such as volatile content, thermal stability, degradation processes, aging, shelf life, sintering behavior, and reaction kinetics of the samples [43].

Figure 6A shows that PPN underwent two stages of degradation. The first stage began at 120.88°C (Td_1_) with negligible mass loss (Δ*w*_1_ = 0.23%), which was attributed to the evaporation of free or adsorbed water. The second main stage occurred at 320.88°C (Td_2_), resulting in a 65.37% mass loss (Δ*w*_2_), associated with the thermal decomposition of the organic structure of piperine. The final residue obtained was 34.4%, indicating the high thermal stability of the compound.

The Np‐AgPPN, represented in Figure 6B, exhibited only one significant degradation stage, beginning at 292.96°C (Td_1_), with a 67.10% mass loss (Δ*w*_1_) and a final residue of 32.9%. The absence of multiple degradation stages suggests that the incorporation of AgNPs modified the thermal degradation process of piperine, promoting its degradation within a narrower temperature range. This behavior indicates a slight reduction in the thermal stability of the compound, likely due to weak chemical interactions between the components, which facilitate the thermal decomposition of the organic matter.

A comparison of the TG/DTG curves revealed that PPN exhibited greater thermal stability, whereas the addition of silver reduced its thermal resistance. According to Pereira et al., the higher percentage of residue obtained for PPN may be related to the formation of additional covalent bonds between matrix constituents. Furthermore, the ∆w observed below 150°C in both samples can be attributed to the evaporation of free and hygroscopic water, as described by Hindi et al. [43].

These results indicate that the incorporation of Ag into PPN negatively affects its thermal stability, promoting faster degradation and lower resistance to temperature increases. DTG analysis confirmed the occurrence of secondary reactions in PPN, which were absent in Np‐AgPPN, demonstrating that modification with AgNPs directly influenced the thermal decomposition of the PPN crystals.

### 3.8 Differential Scanning Calorimetry

**FIGURE 8:**
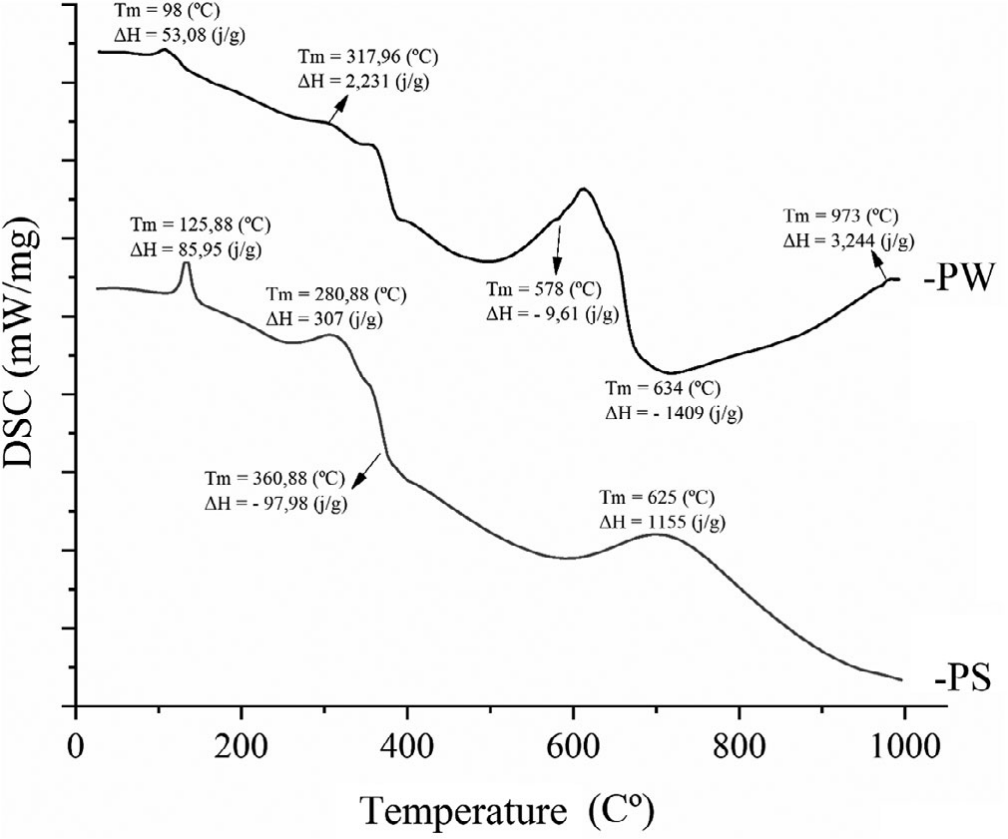
DSC thermograms of PPN and Np‐AgPPN. DSC, differential scanning calorimetry.

DSC was performed to investigate the thermal properties and physical transitions of PPN and NP‐AgPPN samples. Thus, we evaluated the endothermic and exothermic transitions associated with melting, recrystallization, thermal decomposition, and possible intermolecular interactions of the compounds. The obtained thermograms are shown in Figure 8.

The DSC thermograms revealed that PPN has endothermic peaks at 125.88°C and 280.88°C and an exothermic peak at 360.88°C, whereas Np‐AgPPN has multiple endothermic peaks at 98°C and 317.96°C and an exothermic peak at 578°C. For PPN, a pronounced endothermic peak was observed at 125.88°C, with a transition enthalpy Δ*H* = 85.95 J/g, corresponding to the melting point of crystalline piperine. These data are in agreement with the values reported by Ezawa et al. [44], which indicate a melting temperature of piperine between 125°C and 130°C. Similarly, Alves et al. [18] identified a melting peak at approximately 128–130°C. The sample then exhibited a significant endothermic event at 280.88°C (Δ*H* = 307 J/g), which may be related to a structural reorganization or phase transition. An exothermic peak at 360.88°C (Δ*H* = −97.98 J/g) indicates the onset of thermal degradation of the molecule. At higher temperatures, endothermic events at 625°C (Δ*H* = 1155 J/g) were attributed to the burning of carbonaceous residues and the final oxidation of the remaining organic products.

The corresponding curve for Np‐AgPPN revealed distinct thermal behavior with multiple transitions. The first endothermic peak occurred at 98°C (Δ*H* = 53.08 J/g), representing the melting of PPN in a system modified by the presence of silver. A second endothermic event at 317.96°C (Δ*H* = 2.231 J/g) with low enthalpy may indicate a slight structural reorganization, possibly associated with the formation of complexes with silver. The exothermic peak at 578°C (Δ*H* = −9.61 J/g) suggests a partial decomposition stage or exothermic transition induced by the interaction with the metal nanoparticles. At 634°C (Δ*H* = −1409 J/g), a strong exothermic reaction was observed, possibly associated with the complete degradation of the organic matrix and oxidation of the silver compound. Finally, the event at 973°C (Δ*H* = 3.244 J/g) may indicate the sintering or reorganization of metallic residues, which is characteristic of silver‐containing compounds [45].

These results indicate that the complexation of PNN with Ag significantly altered its thermal profile, reducing its initial melting temperature and introducing new thermal events. The diversity of transitions observed in PNN reinforces the hypothesis of complex molecular interactions, such as the formation of inclusion complexes or redox reactions. However, further thermal analyses are recommended to elucidate the thermodynamic mechanisms involved and confirm the formation of new silver‐induced structural arrangements.

### 3.9 In Vitro Cytotoxicity

PPN is a bioactive alkaloid found in *P. nigrum* that has several pharmacological activities, including antioxidant, anti‐inflammatory, and antitumor effects. However, its association with AgNPs can significantly alter its bioavailability, toxicity, and cell interaction profiles. Therefore, investigating the effects of PPN and Np‐AgPPN on cell viability and cytotoxicity is essential for assessing the safety and therapeutic potential of these compounds.

In vitro cell viability analysis revealed the cytotoxic effects of these compounds on specific cell lines, contributing to the validation of their potential biomedical applications. The in vitro cytotoxicity of the compounds was evaluated in three gastric tumor cell lines and a nonneoplastic human kidney cell line using the MTT assay. The results are presented in Table 4.

**TABLE 4:** Mean inhibitory concentration (IC_50_) values for cell growth, in µg/mL, after 72 h of incubation, obtained by the MTT assay.

Compounds	AGP01	AGP01 PIWIL1^−^/^−^	ACP02	HEK‐293
PPN	50.7 *R* ^2^ = 0.833	50.3 *R* ^2^ = 0.907	55.7 *R* ^2^ = 0.911	51.7 *R* ^2^ = 0.928
Selective index (SI)	1.1	1.0	0.9	—
Np‐AgPPN	23.6 (20.1–28.5) *R* ^2^ = 0.904	23.1 (20.2–26.6) *R* ^2^ = 0.973	32.5 (29.3–36.2) *R* ^2^ = 0.976	33.9 (29.5–42.6) *R* ^2^ = 0.968
Selective index (SI)	1.4	1.5	1.0	—

On the basis of the dose–response curve analysis, it was possible to estimate the IC_50_ values for each compound. The PPN compound exhibited IC_50_ values ranging from 50.3 to 55.7 µg/mL after 72 h of exposure, indicating cytotoxicity against the tested gastric cancer cells. This result corroborates previous studies reporting the cytotoxic activity of PPN in different tumor types [46, 47]. In contrast, Np‐AgPPN presented considerably lower IC_50_ values, ranging from 23.1 to 33.9 µg/mL. Accordingly, Np‐AgPPN demonstrated a more pronounced activity against gastric cancer cells compared with isolated PPN, suggesting that the incorporation of silver enhances the cytotoxic effect of PPN.

Metallic silver and its associated ions have been reported to intensify oxidative stress, impair mitochondrial integrity, and disrupt redox homeostasis [48, 49]. When these processes occur in combination with bioactive molecules such as PPN, they can lead to a cytotoxic overload in tumor cells. The enhanced activity observed for Np‐AgPPN suggests that the conjugated system generates a highly reactive local microenvironment, thereby promoting cell death mechanisms already described for both components when used individually [50, 51].

The enhancement in cytotoxic activity observed for Np‐AgPPN suggests a functional interaction between silver and PPN, potentially through synergistic amplification of cellular damage. This interpretation is consistent with studies describing the cytotoxic effects of silver nanoparticles, particularly in cells with high oxidative metabolism, such as gastric cancer cells [52].

**FIGURE 9:**
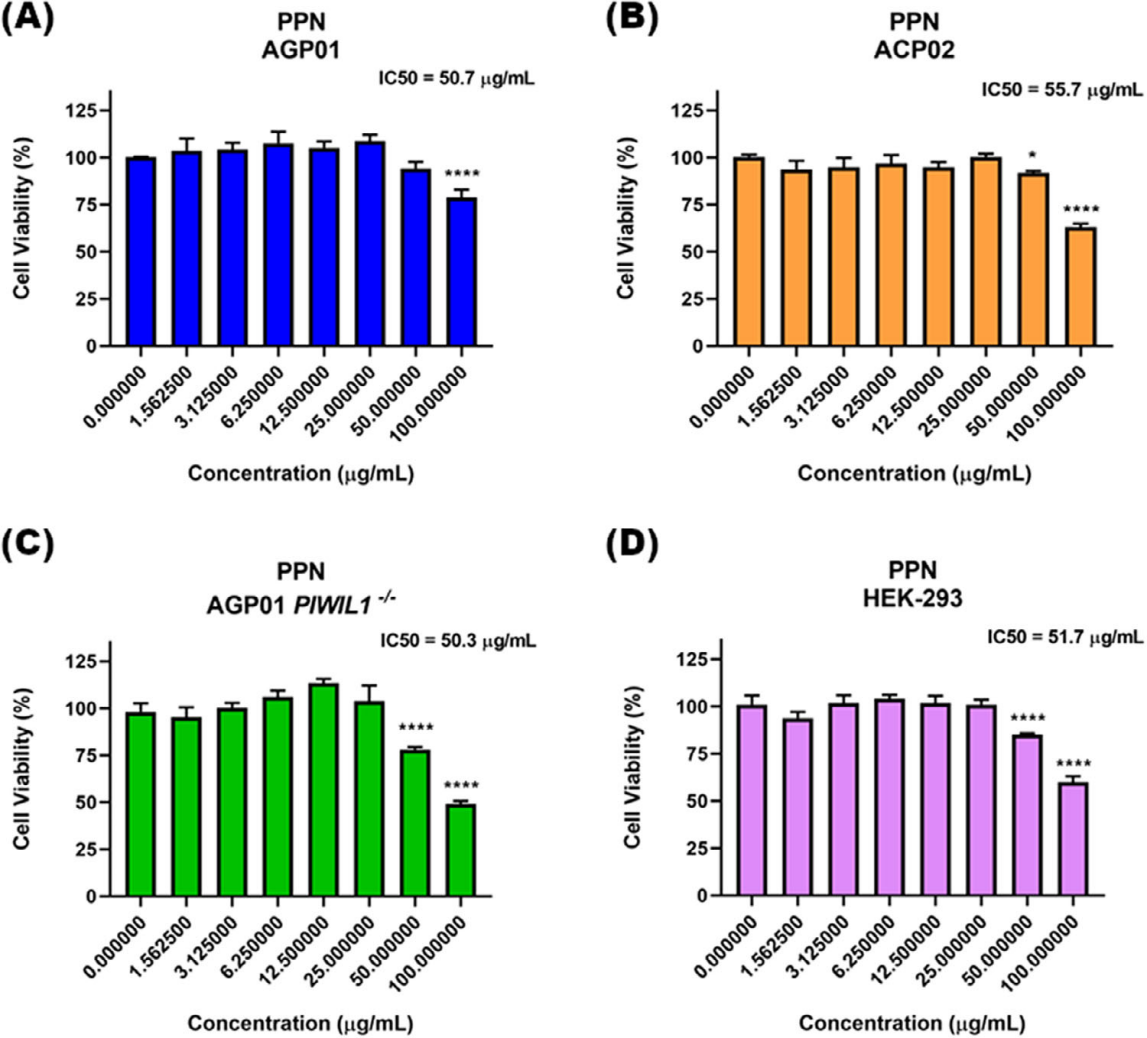
Effect of piperine (PPN) on cell viability after 72 h of treatment, as evaluated by the MTT assay. (A) AGP01 – gastric ascites adenocarcinoma cell line; (B) AGP01 PIWIL1^−^/^−^ – gastric ascites adenocarcinoma cell line with PIWIL1 gene inactivation; (C) ACP02 – primary diffuse‐type gastric adenocarcinoma cell line; (D) HEK‐293 – non‐neoplastic human embryonic kidney cell line. Results are expressed as mean ± standard deviation (n = 3) and presented as percentage of viability relative to the negative control. ^***^p < 0.001; ^****^p < 0.0001.

Analysis of cell viability showed that relatively high concentrations of PPN were required to reduce cell survival, with significant inhibition occurring only at the two highest concentrations tested (50 and 100 µg/mL), although cytotoxic effects were observed across all evaluated cell lines (Figure 9). In accordance with this profile, the IC_50_ values for PPN remained consistent at approximately 50 µg/mL, indicating a moderate level of cytotoxic induction.

**FIGURE 10:**
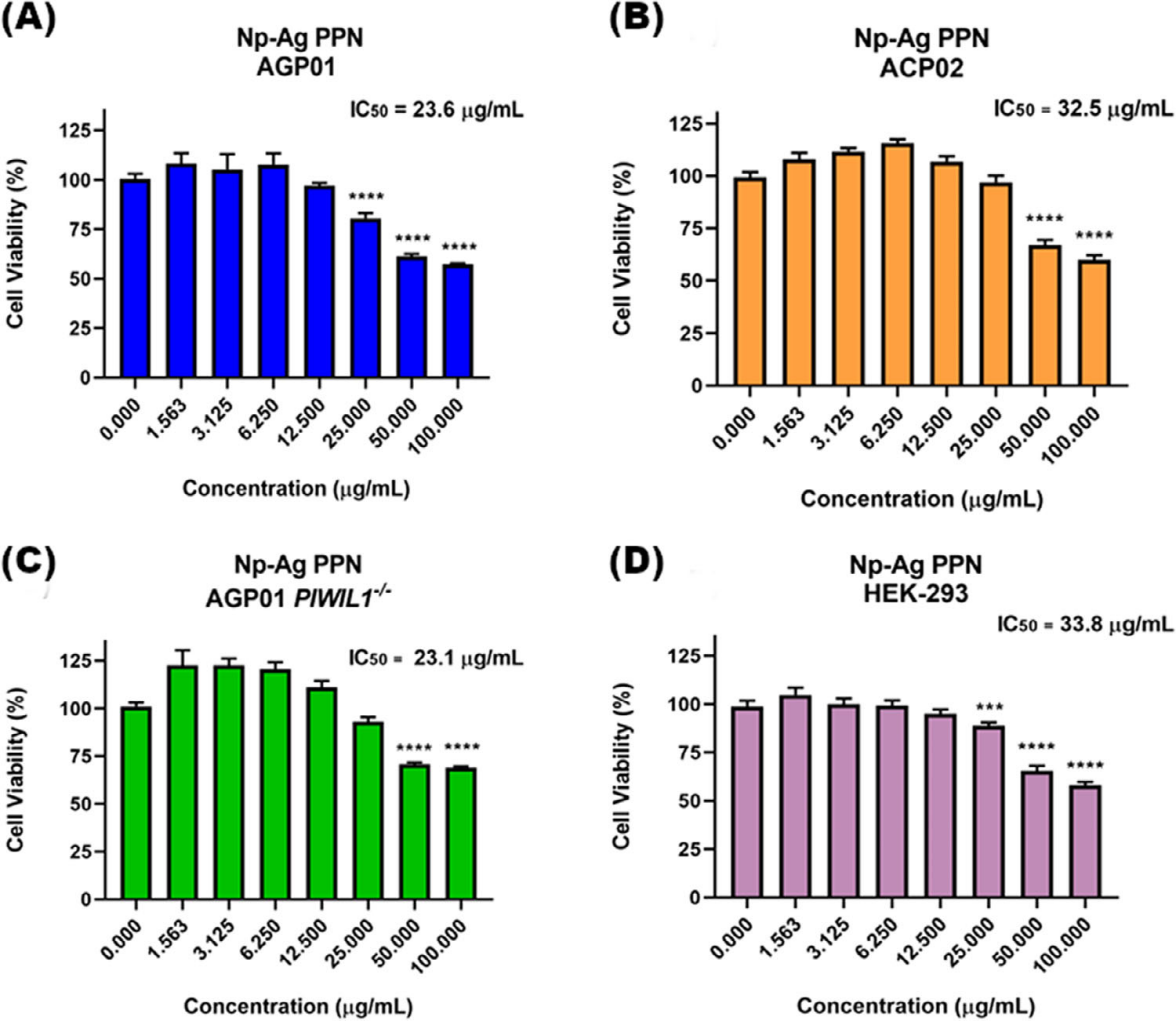
Effect of piperine‐functionalized silver nanoparticles (Np‐AgPPN) on cell viability after 72 h of treatment, as evaluated by the MTT assay. (A) AGP01 – gastric ascites adenocarcinoma cell line; (B) AGP01 PIWIL1^−^/^−^ – gastric ascites adenocarcinoma cell line with PIWIL1 gene inactivation; (C) ACP02 – primary diffuse‐type gastric adenocarcinoma cell line; (D) HEK‐293 – non‐neoplastic human embryonic kidney cell line. Results are expressed as mean ± standard deviation (n = 3) and presented as percentage of cell viability relative to the negative control. ^***^p < 0.001; ^****^p < 0.0001.

In contrast, the results obtained for Np‐AgPPN (Figure 10) demonstrated cytotoxic activity in all analyzed cell lines, with notable potency in the AGP01 PIWIL1^−^/^−^ gastric cancer line, which exhibited the lowest IC_50_ value (23.1 µg/mL). Np‐AgPPN also showed substantial activity in the AGP01 and ACP02 gastric cancer cell lines, with mean IC_50_ values of approximately 26 µg/mL. Beyond its ability to reduce cell viability at lower concentrations (25 µg/mL), these findings reinforce that conjugation of silver to PPN enhances its antiproliferative activity. Regarding the non‐tumor cell line (HEK‐293), cytotoxic activity was observed with an IC_50_ of 33.9 µg/mL. However, this value was approximately 1.5 times higher than that observed in gastric tumor cell lines, demonstrating favorable selectivity.

Recent evidence indicates that silver nanoparticles exert cytotoxicity primarily through increased generation of reactive oxygen species (ROS), mitochondrial dysfunction, and activation of cell death pathways. Miranda et al. [53] and Bin‐Jumah et al. [54] showed that AgNPs promote the accumulation of cytoplasmic and mitochondrial ROS, resulting in lipid peroxidation, DNA damage, and caspase activation. In parallel, PPN is also known to modulate cellular redox balance by elevating ROS levels. In colorectal cancer cells, piperine induced a significant rise in intracellular ROS derived from multiple sources, triggering apoptotic signaling and cell cycle arrest [55].

In a gastric cancer cell line (HGC‐27), piperine has also been shown to increase ROS levels and induce mitochondrial damage, leading to the activation of pro‐apoptotic proteins such as Bax, release of cytochrome c, and subsequent caspase activation [56]. Similarly, in oral carcinoma cells, piperine triggered a dose‐dependent increase in ROS, loss of mitochondrial membrane potential, and activation of caspase‐3, ultimately culminating in apoptosis [57].

These previously described mechanisms for both compounds provide a plausible explanation for the lower IC_50_ values observed for Np‐AgPPN in the present study, supporting the consistency of our findings with the established scientific literature. The difference between the IC_50_ values of isolated PPN and Np‐AgPPN suggests a potential synergistic effect that enhances cytotoxic activity. Moreover, the conjugated system appears to confer greater stability and promote the internalization of PPN by tumor cells, further contributing to the increased cytotoxic response.

Given this context, the results presented here are consistent with the increasing recognition that silver nanoparticles constitute an efficient platform for therapeutically exploiting oxidative stress in tumors characterized by highly reactive metabolic profiles. The enhanced cytotoxic activity of PPN following its incorporation into AgNPs further underscores the potential of nanoparticle–phytochemical hybrid systems as promising candidates in the development of novel antitumor strategies.

The biological activity exhibited by Np‐AgPPN highlights the need for continued investigation to elucidate the mechanisms through which this conjugated system exerts its cytotoxic effects. A detailed understanding of how the compound interacts with cellular pathways and the specific role of Ag in its activity is essential for developing new therapies based on this conjugated compound.

## 4 Conclusions

This study reports the formation of AgNPs using a low‐cost, simple, rapid, and environmentally friendly approach based on a piperine‐rich *P. nigrum* seed extract (PPN). Colorimetric, spectroscopic (UV–Vis and FTIR‐ATR), morphological (SEM–EDS), structural (XRD), and thermal (TG/DTG and DSC) analyses revealed significant physicochemical changes in PPN following silver incorporation. Np‐AgPPN exhibited superior cytotoxic activity compared to PPN against gastric cancer cell lines, including AGP‐01 (gastric ascites), AGP‐01 PIWIL1^−^/^−^ (gastric ascites with inactivated PIWIL1 gene), and ACP02 (primary gastric adenocarcinoma of the diffuse type). Thus, the findings of this study demonstrate that functionalization of piperine with AgNPs not only alters its physicochemical properties but also enhances its biological activity, highlighting Np‐AgPPN as a promising system for therapeutic applications, particularly in the context of gastric cancer. Nevertheless, the present study is limited to in vitro evaluations, and further in vivo investigations are required to confirm the safety, efficacy, and pharmacokinetic behavior of this nanosystem. In addition, future studies should address long‐term stability, reproducibility, and scalability of the green synthesis process to support potential translational and industrial applications. A deeper investigation of the molecular mechanisms underlying the enhanced cytotoxicity is also warranted to fully elucidate the therapeutic potential of piperine‐functionalized AgNPs.

## Conflicts of Interest

The authors declare no conflicts of interest.

## Data Availability

The data that support the findings of this study are available from the corresponding author upon reasonable request.

## References

[1] M. A. Zarbin , “The Nanotechnology Revolution,” Asia‐Pacific Journal of Ophthalmology 3 (2014): 131–132, 10.1097/APO.0000000000000064. 26107580

[2] D. R. Boverhof , C. M. Bramante , J. H. Butala , et al., “Comparative Assessment of Nanomaterial Definitions and Safety Evaluation Considerations,” Regulatory Toxicology and Pharmacology: RTP 73 (2015): 137–150, 10.1016/j.yrtph.2015.06.001. 26111608

[3] A. Dhaka , S. Chand Mali , S. Sharma , and R. Trivedi , “A Review on Biological Synthesis of Silver Nanoparticles and Their Potential Applications,” Results in Chemistry 6 (2023): 101108, 10.1016/j.rechem.2023.101108.

[4] N. S. Alharbi , N. S. Alsubhi , and A. I. Felimban , “Green Synthesis of Silver Nanoparticles Using Medicinal Plants: Characterization and Application,” Journal of Radiation Research and Applied Sciences 15 (2022): 109–124, 10.1016/j.jrras.2022.06.012.

[5] R. Abbas , J. Luo , X. Qi , et al., “Silver Nanoparticles: Synthesis, Structure, Properties and Applications,” Nanomaterials 14 (2024): 1425, 10.3390/nano14171425. 39269087 PMC11397261

[6] H. Duman , F. Eker , E. Akdaşçi , A. M. Witkowska , M. Bechelany , and S. Karav , “Silver Nanoparticles: A Comprehensive Review of Synthesis Methods and Chemical and Physical Properties,” Nanomaterials 14 (2024): 1527, 10.3390/nano14181527. 39330683 PMC11434896

[7] M. H. Sarfraz , M. Zubair , B. Aslam , et al., “Comparative Analysis of Phyto‐Fabricated Chitosan, Copper Oxide, and Chitosan‐Based CuO Nanoparticles: Antibacterial Potential Against *Acinetobacter baumannii* Isolates and Anticancer Activity Against HepG2 Cell Lines,” Frontiers in Microbiology 14 (2023): 1188743, 10.3389/fmicb.2023.1188743. 37323910 PMC10264586

[8] S. Dawadi , S. Katuwal , A. Gupta , et al., “Current Research on Silver Nanoparticles: Synthesis, Characterization, and Applications,” Journal of Nanomaterials 2021 (2021): 6687290, 10.1155/2021/6687290.

[9] S. Muzammil , J. Neves Cruz , R. Mumtaz , et al., “Effects of Drying Temperature and Solvents on In Vitro Diabetic Wound Healing Potential of *Moringa oleifera* Leaf Extracts,” Molecules (Basel, Switzerland) 28 (2023): 710, 10.3390/molecules28020710. 36677768 PMC9864430

[10] M. Ognjanović , K. Nikolić , M. Radenković , A. Lolić , D. Stanković , and S. Živković , “Picosecond Laser‐Assisted Synthesis of Silver Nanoparticles With High Practical Application as Electroanalytical Sensor, Surfaces and Interfaces,” Surfaces and Interfaces 35 (2022): 102464, 10.1016/j.surfin.2022.102464.

[11] A. Roy , O. Bulut , S. Some , A. K. Mandal , and M. D. Yilmaz , “Green Synthesis of Silver Nanoparticles: Biomolecule‐Nanoparticle Organizations Targeting Antimicrobial Activity,” RSC Advances 9 (2019): 2673–2702, 10.1039/c8ra08982e. 35520490 PMC9059941

[12] S. Korpayev , H. Hamrayev , N. Aničić , et al., “Green Synthesis of Silver Nanoparticles With *Alhagi persarum* Flowers Extract and Its Antioxidant, Antimicrobial, and Cytotoxic Activities,” Biomass Convers Biorefinery 14 (2024): 24715–24729, 10.1007/s13399-023-04648-1.

[13] K. D. S. M. Mesquita , B. S. de Feitosa , J. N. Cruz , et al., “Chemical Composition and Preliminary Toxicity Evaluation of the Essential Oil From *Peperomia circinnata* Link Var. Circinnata. (Piperaceae) in *Artemia salina* Leach,” Molecules (Basel, Switzerland) 26 (2021): 7359, 10.3390/molecules26237359. 34885940 PMC8659193

[14] Z. Ferdous and A. Nemmar , “Health Impact of Silver Nanoparticles: A Review of the Biodistribution and Toxicity Following Various Routes of Exposure,” International Journal of Molecular Sciences 21 (2020): 2375, 10.3390/ijms21072375. 32235542 PMC7177798

[15] S. Korpayev , A. Karakeçili , H. Dumanoğlu , and S. Ibrahim Ahmed Osman , “Chitosan and Silver Nanoparticles Are Attractive Auxin Carriers: A Comparative Study on the Adventitious Rooting of Microcuttings in Apple Rootstocks,” Biotechnology Journal 16 (2021): e2100046, 10.1002/biot.202100046. 34028191

[16] S. Antunes Filho , M. S. dos Santos , O. A. L. dos Santos , et al., “Biosynthesis of Nanoparticles Using Plant Extracts and Essential Oils,” Molecules (Basel, Switzerland) 28 (2023): 3060, 10.3390/molecules28073060. 37049821 PMC10095647

[17] Z. A. Omar , R. S. Abduljabar , S. M. Sajadi , S. A. Mahmud , and R. O. Yahya , “Recent Progress in Eco‐Synthesis of Essential Oil‐Based Nanoparticles and Their Possible Mechanisms,” Industrial Crops and Products 187 (2022): 115322, 10.1016/j.indcrop.2022.115322.

[18] F. S. Alves , J. N. Cruz , I. N. de Farias Ramos , et al., “Evaluation of Antimicrobial Activity and Cytotoxicity Effects of Extracts of *Piper nigrum* L. and Piperine,” Separations 10 (2023): 21, 10.3390/separations10010021.

[19] F. S. Alves , J. A. de Rodrigues Do Rego , M. L. Da Costa , et al., “Spectroscopic Methods and In Silico Analyses Using Density Functional Theory to Characterize and Identify Piperine Alkaloid Crystals Isolated From Pepper (*Piper nigrum* L.),” Journal of Biomolecular Structure & Dynamics 38 (2020): 2792–2799, 10.1080/07391102.2019.1639547. 31282297

[20] I. N. F. de Ramos , M. F. da Silva , J. M. S. Lopes , et al., “Extraction, Characterization, and Evaluation of the Cytotoxic Activity of Piperine in Its Isolated Form and in Combination With Chemotherapeutics Against Gastric Cancer,” Molecules (Basel, Switzerland) 28 (2023): 5587, 10.3390/molecules28145587. 37513459 PMC10385350

[21] M. F. Leal , J. L. Martins do Nascimento , C. E. A. da Silva , et al., “Establishment and Conventional Cytogenetic Characterization of Three Gastric Cancer Cell Lines,” Cancer Genetics and Cytogenetics 195 (2009): 85–91, 10.1016/j.cancergencyto.2009.04.020. 19837275

[22] T. Araújo , A. Khayat , L. Quintana , et al., “Piwi Like RNA‐Mediated Gene Silencing 1 Gene as a Possible Major Player in Gastric Cancer,” World Journal of Gastroenterology 24 (2018): 5338–5350, 10.3748/wjg.v24.i47.5338. 30598579 PMC6305533

[23] R. Mirzayans , B. Andrais , and D. Murray , “Viability Assessment Following Anticancer Treatment Requires Single‐Cell Visualization,” Cancers (Basel) 10 (2018): 255, 10.3390/cancers10080255. 30071623 PMC6115892

[24] G. Shumi , T. B. Demissie , R. Eswaramoorthy , R. F. Bogale , G. Kenasa , and T. Desalegn , “Biosynthesis of Silver Nanoparticles Functionalized With Histidine and Phenylalanine Amino Acids for Potential Antioxidant and Antibacterial Activities,” ACS Omega 8 (2023): 24371–24386, 10.1021/acsomega.3c01910. 37457474 PMC10339392

[25] C. A. Demarchi , A. B. Cruz , C. M. da Silva Bitencourt , et al., “ *Eugenia umbelliflora* Mediated Reduction of Silver Nanoparticles Incorporated Into O‐Carboxymethylchitosan/y‐Fe_2_O_3_: Synthesis, Antimicrobial Activity and Toxicity,” International Journal of Biological Macromolecules 155 (2020): 614–624, 10.1016/j.ijbiomac.2020.03.247. 32246959

[26] J. Pathak , A. S. Sonker , V. S. Rajneesh , D. Kumar , and R. P. Sinha , “Synthesis of Silver Nanoparticles From Extracts of *Scytonema geitleri* HKAR‐12 and Their In Vitro Antibacterial and Antitumor Potentials,” Letters in Applied NanoBioScience 8 (2019): 576–585, 10.33263/LIANBS83.576585.

[27] A. Jangchud and M. S. Chinnan , “Peanut Protein Film as Affected by Drying Temperature and pH of Film Forming Solution,” Journal of Food Science 64 (1999): 153–157, 10.1111/j.1365-2621.1999.tb09881.x.

[28] L. Sulman , “Isolation of Piperine From Black Pepper (*Piper nigrum*) in the Provision of Standard Compounds for Natural Chemical Practice and Research Activities,” Journal Pijar MIPA 16 (2021): 683–687, 10.29303/jpm.v16i5.2981.

[29] L. Gorgani , M. Mohammadi , G. D. Najafpour , and M. Nikzad , “Sequential Microwave‐Ultrasound‐Assisted Extraction for Isolation of Piperine From Black Pepper (*Piper nigrum* L.),” Food and Bioprocess Technology 10 (2017): 2199–2207, 10.1007/s11947-017-1994-0.

[30] S. Bahri , Y. Ambarwati , M. Iqbal , and A. A. Baihaqy , “Synthesis 4‐Piperoilmorpholine From Piperine,” Journal of Physics: Conference Series 1338 (2019): 12010, 10.1088/1742-6596/1338/1/012010.

[31] N. Kusumorini , A. K. Nugroho , S. Pramono , and R. Martien , “Development of New Isolation and Quantification Method of Piperine From White Pepper Seeds (*Piper nigrum* L) Using a Validated HPLC,” Indonesian Journal of Pharmacy 32 (2021): 158–165, 10.22146/ijp.866.

[32] R. L. Shriner , R. C. Fuson , D. Y. Curtin , and T. C. Morrill , The Systematic Identification of Organic Compounds, 6th ed. (John Wiley & Sons, 1980), pp. 318–363.

[33] G. V. da Silva Pereira , G. V. da Silva Pereira , E. M. P. Xavier Neves , et al., “Effect of the Mixture of Polymers on the Rheological and Technological Properties of Composite Films of Acoupa Weakfish (*Cynoscion acoupa*) and Cassava Starch (*Manihot esculenta* C.),” Food and Bioprocess Technology 14 (2021): 1199–1215, 10.1007/s11947-021-02622-1.

[34] C. Kucuk , S. Yurdakul , N. Özdemir , and B. Erdem , “Crystal Structure, Vibrational Spectroscopy, 1H NMR, and DFT Analyses With Antibacterial Activity Studies on Silver Nitrate Complex of 5‐Iodoindole,” Inorganic Chemistry Communications 150 (2023): 110465, 10.1016/j.inoche.2023.110465.

[35] M. Xu , J. Wang , C. Li , J. Zhang , Y. Liu , and G. Yuan , “Effect of Electric Field to Infrared Absorption Properties of Sodium Nitrate on Silver/Diamond Powder (Ag/DP) Composites,” Chemical Physics Letters 613 (2014): 10–14, 10.1016/j.cplett.2014.08.046.

[36] K. V. Padalkar and V. G. Gaikar , “Extraction of Piperine From *Piper nigrum* (Black Pepper) by Aqueous Solutions of Surfactant and Surfactant + Hydrotrope Mixtures,” Separation Science and Technology 43 (2008): 3097–3118, 10.1080/01496390802063887.

[37] J. Tang , X. Liu , Y. Ge , and F. Wang , “Silver Nanoparticle‐Anchored Human Hair Kerateine/PEO/PVA Nanofibers for Antibacterial Application and Cell Proliferation,” Molecules (Basel, Switzerland) 26 (2021): 2783, 10.3390/molecules26092783. 34066875 PMC8125921

[38] A. Adan , Y. Kiraz , and Y. Baran , “Cell Proliferation and Cytotoxicity Assays,” Current Pharmaceutical Biotechnology 17 (2016): 1213–1221, 10.2174/1389201017666160808160513. 27604355

[39] R. A. Khan , “Natural Products Chemistry: The Emerging Trends and Prospective Goals, Saudi,” Pharmaceutical Journal 26 (2018): 739–753, 10.1016/j.jsps.2018.02.015. PMC6036106 29991919

[40] A. Stasiłowicz , N. Rosiak , E. Tykarska , et al., “Combinations of Piperine with Hydroxypropyl‐β‐Cyclodextrin as a Multifunctional System,” International Journal of Molecular Sciences 22 (2021): 4195, 10.3390/ijms22084195. 33919582 PMC8072981

[41] M. K. Saini , S. N. Sanyal , and K. Vaiphei , “Piroxicam and C‐Phycocyanin Mediated Apoptosis in 1,2‐Dimethylhydrazine Dihydrochloride Induced Colon Carcinogenesis: Exploring the Mitochondrial Pathway,” Nutrition and Cancer 64 (2012): 409–418, 10.1080/01635581.2012.655402. 22369161

[42] T. Le Na Nguyen , N. Huyen Nguyen , and C. Doanh Sai , et al., “Development and Characterization of Gelatin Microgel‐Stabilized Silver Nanoparticles Loaded With Curcumin: Evaluation of Antibacterial, Antioxidant, and Antiproliferative Activities,” Science Progress 108 (2025): 00368504251338628, 10.1177/00368504251338628. 40296548 PMC12041703

[43] S. S. Z. Hindi , M. O. Albureikan , A. A. Al‐Ghamdi , H. Alhummiany , and M. S. Ansari , “Synthesis, Characterization and Biodegradation of Gum Arabic‐Based Bioplastic Membranes,” Nanoscience and Nanotechnology 4 (2017): 32–42, 10.12691/nnr-4-2-1.<.

[44] T. Ezawa , Y. Inoue , S. Tunvichien , R. Suzuki , and I. Kanamoto , “Changes in the Physicochemical Properties of Piperine/β‐Cyclodextrin due to the Formation of Inclusion Complexes,” International Journal of Medicinal Chemistry 2016 (2016): 1–9, 10.1155/2016/8723139. PMC4779834 26998357

[45] S. Ali , P. Saokaew , A. Aman , et al., “Enhancing Solubility and Stability of Piperine Using β‐Cyclodextrin Derivatives: Computational and Experimental Investigations,” Journal of Biomolecular Structure & Dynamics 43 (2025): 2596–2609, 10.1080/07391102.2024.2305696. 38260962

[46] E. Turrini , P. Sestili , and C. Fimognari , “Overview of the Anticancer Potential of the “King of Spices” *Piper nigrum* and Its Main Constituent Piperine,” Toxins (Basel) 12 (2020): 747, 10.3390/toxins12120747. 33256185 PMC7761056

[47] L. P. Cardoso , S. O. de Sousa , J. P. Gusson‐Zanetoni , et al., “Piperine Reduces Neoplastic Progression in Cervical Cancer Cells by Downregulating the Cyclooxygenase 2 Pathway,” Pharmaceuticals 16 (2023): 103, 10.3390/ph16010103. 36678600 PMC9866887

[48] P. Takáč , R. Michalková , M. Čižmáriková , Z. Bedlovičová , Ľ. Balážová , and G. Takáčová , “The Role of Silver Nanoparticles in the Diagnosis and Treatment of Cancer: Are There any Perspectives for the Future?,” Life 13 (2023): 466, 10.3390/life13020466. 36836823 PMC9965924

[49] M. M. Rohde , C. M. Snyder , J. Sloop , et al., “The Mechanism of Cell Death Induced by Silver Nanoparticles Is Distinct From Silver Cations,” Particle and Fibre Toxicology 18 (2021): 37, 10.1186/s12989-021-00430-1. 34649580 PMC8515661

[50] A. Raj , L. Vidya , A. Reji , S. Neelima , V. M. Aparna , and C. Sudarsanakumar , “Piperonal‐Coated Silver Nanoparticles: A Study on Cytotoxicity and Protein Binding,” Spectrochimica Acta, Part A: Molecular and Biomolecular Spectroscopy 334 (2025): 125890, 10.1016/j.saa.2025.125890. 39987606

[51] G. Raja , Y.‐K. Jang , J.‐S. Suh , H.‐S. Kim , S. H. Ahn , and T.‐J. Kim , “Microcellular Environmental Regulation of Silver Nanoparticles in Cancer Therapy: A Critical Review,” Cancers (Basel) 12 (2020): 664, 10.3390/cancers12030664. 32178476 PMC7140117

[52] A. Moshrefi and S. M. Hosseini , “In Vitro and In Vivo Evaluation of Anti‐Tumorigenesis Potential of Nano Silver for Gastric Cancer Cells,” Journal of Molecular Histology 56 (2024): 14, 10.1007/s10735-024-10315-0. 39611988

[53] R. R. Miranda , I. Sampaio , and V. Zucolotto , “Exploring Silver Nanoparticles for Cancer Therapy and Diagnosis,” Colloids Surfaces B Biointerfaces 210 (2022): 112254, 10.1016/j.colsurfb.2021.112254. 34896692

[54] M. Bin‐Jumah , M. AL‐Abdan , G. Albasher , and S. Alarifi , “Effects of Green Silver Nanoparticles on Apoptosis and Oxidative Stress in Normal and Cancerous Human Hepatic Cells In Vitro,” International Journal of Nanomedicine 15 (2020): 1537–1548, 10.2147/ijn.s239861. 32210550 PMC7074819

[55] W.‐L. Chang , J.‐Y. Peng , C.‐L. Hong , et al., “Piperine Induces Apoptosis and Cell Cycle Arrest via Multiple Oxidative Stress Mechanisms and Regulation of PI3K/Akt and MAPK Signaling in Colorectal Cancer Cells,” Antioxidants 14 (2025): 892, 10.3390/antiox14070892. 40722996 PMC12292380

[56] L. Guo , Y. Yang , Y. Sheng , J. Wang , S. Ruan , and C. Han , “Mechanism of Piperine in Affecting Apoptosis and Proliferation of Gastric Cancer Cells via ROS‐Mitochondria‐Associated Signalling Pathway,” Journal of Cellular and Molecular Medicine 25 (2021): 9513–9522, 10.1111/jcmm.16891. 34464498 PMC8505830

[57] S. Siddiqui , M. S. Ahamad , A. Jafri , M. Afzal , and M. Arshad , “Piperine Triggers Apoptosis of Human Oral Squamous Carcinoma Through Cell Cycle Arrest and Mitochondrial Oxidative Stress,” Nutrition and Cancer 69 (2017): 791–799, 10.1080/01635581.2017.1310260. 28426244

